# An Embodied Perspective as a Victim of Sexual Harassment in Virtual Reality Reduces Action Conformity in a Later Milgram Obedience Scenario

**DOI:** 10.1038/s41598-020-62932-w

**Published:** 2020-04-10

**Authors:** Solène Neyret, Xavi Navarro, Alejandro Beacco, Ramon Oliva, Pierre Bourdin, Jose Valenzuela, Itxaso Barberia, Mel Slater

**Affiliations:** 1 Event Lab, Department of Clinical Psychology and Psychobiology, University of Barcelona, Barcelona, Spain; 2 Department of Cognition, Development and Educational Psychology, University of Barcelona, Barcelona, Spain; 3 Institute of Neurosciences of the University of Barcelona, Barcelona, Spain; 4 Present Address: Computer Science, Multimedia and Telecommunications Department, Universitat Oberta de Catalunya, Barcelona, Spain

**Keywords:** Psychology, Mathematics and computing

## Abstract

Group pressure can often result in people carrying out harmful actions towards others that they would not normally carry out by themselves. However, few studies have manipulated factors that might overcome this. Here male participants (n = 60) were in a virtual reality (VR) scenario of sexual harassment (SH) of a lone woman by a group of males in a bar. Participants were either only embodied as one of the males (Group, n = 20), or also as the woman (Woman, n = 20). A control group (n = 20) only experienced the empty bar, not the SH. One week later they were the Teacher in a VR version of Milgram’s Obedience experiment where they were encouraged to give shocks to a female Learner by a group of 3 virtual males. Those who had been in the Woman condition gave about half the number of shocks of those in the Group condition, with the controls between these two. We explain the results through embodiment promoting identification with the woman or the group, and delegitimization of the group for those in the Woman condition. The experiment raised important ethical issues, showing that a VR study with positive ethical intentions can sometimes produce unexpected and non-beneficent results.

## Introduction

Group pressure and the need to conform can result in significant distortions of an individual’s judgment and decision making – a well-known case by Asch^1^ being misjudgement of geometric shapes under peer pressure. However, Stanley Milgram argued for the distinction between signal conformity and action conformity^2,3^. In Asch’s experiment people only verbally signalled conformity with the group but this had no further consequences (signal conformity). In contrast, in one experiment described by Milgram, participants were influenced to (apparently) administer electric shocks of greater and increasing voltage to a stranger at the behest of two confederates who demanded this, supposedly as a learning experiment. This is an example of action conformity since the behaviour of the subject would cause pain to another person. When there were no confederates, participants tended to choose the lowest shocks possible. Taking this out of the lab to real situations, conformity to group pressure can result in people engaging in evil acts that they would not normally do themselves individually, such as in the Stanford Prison Experiments^4^ – even to the extent of taking part in mass killings – e.g., Stammers^5^, Chapter 18.

The most salient finding of the obedience experiments has been that a surprisingly high proportion of people will administer apparently lethal shocks to a stranger at the behest of an authority figure. This was originally interpreted by Milgram as caused by their obedience to authority. However, alternative explanations have gained currency. Haslam, *et al*.^6^ carried out an analysis of the results of all of Milgram’s experiments, comprising 21 different conditions, and found an overall obedience level of 44%. A meta-analysis of the results of all the experiments concluded that the likelihood of obedience was positively correlated with the directiveness of the experimenter, and negatively related with several factors: the pressure of a peer group to disobey, the distance between the Teacher and Learner and how direct their interactions were, the distance between the Teacher and Experimenter, the intimacy between Teacher and Learner, and the illegitimacy and inconsistency of the experimenter.

The factors elicited above were found to contribute to the extent of obedience or disobedience. Other research, however, has reframed the Milgram paradigm away from issues of obedience towards shared social identity and ‘followership’^7–10^. In other words, if subjects identify with the experimenter or the goals of the experimenter, and think that what is happening is right, then they will be more likely to administer the shocks. This has been shown, for example, in the willingness of subjects to administer the shocks for the sake of science, rather than because they were simply obeying authority^11^. A virtual reality (VR) study has also lent weight to this idea, in addition showing that in spite of giving shocks subjects were nevertheless concerned for the virtual Learner^12^.

The experiment described in this paper was designed to explore whether taking the embodied perspective of a woman subject to harassment by a group of men would later diminish the number of shocks they would administer in the Milgram paradigm, compared to other conditions. In its simplest form our question was how their action conformity in the Obedience scenario would be modulated if one week earlier they had been in the embodied perspective of a woman on the receiving end of verbal sexual harassment by the same group of virtual men acting as experimenters.

The focus, therefore, was on giving men the experience of being in the situation of a woman being harassed, and we used the Milgram paradigm as an objective measure of whether that intervention had any influence on their subsequent aggressive behaviour. Another way to put this is that the purpose was to see whether embodiment as the woman would break the in-group solidarity with the virtual males, that is, the ‘followership’ of those male experimenters. A third way to look at this is that embodiment as the woman a week before the Milgram scenario would be a salient way of minimising the psychological distance between the participant (Teacher) and Learner. Next we describe what we mean by ‘embodiment’.

In VR it is possible to substitute a person’s body by a life-sized manikin body^13^ or virtual body^14^ that is apparently spatially coincident with their real body. Using real-time motion capture the virtual body can be programmed to move in synchrony and correspondence with the participant’s real body movements. This setup can give rise to the perceptual illusion of ownership over the virtual body, a paradigm based on the rubber hand illusion^15^. Virtual body ownership can also result in changes in attitudes and behaviours of the experiencer, with respect to age^16–18^, race^19–21^, pain sensitivity^22,23^, motor behaviour^24^, and cognitive task performance^25^.

There are three relevant studies where embodiment takes place in the context of a negative social interaction. Groom, *et al*.^26^ embodied White people in a Black virtual body in the context of a job interview – a situation known to exacerbate implicit bias against Black. It was found that the level of implicit bias against Black people increased as a result of this intervention, in contrast to the studies on racial bias mentioned above. Hamilton-Giachritsis, *et al*.^27^ embodied mothers in a body representing a child of about 4 years old. They then had an interaction with a virtual mother who displayed positive attitudes towards the child, or negative and critical attitudes. Interaction with the negative mother increased their stress levels, enhanced their fear of violence, but also enhanced empathy towards their own children. Seinfeld, *et al*.^28^ embodied male domestic violence offenders, and a group of male controls (non-offenders), in the virtual body of a woman who was then subject to an abusive attack from a male virtual character. It was found that the prior deficit of offenders in recognising fear in the faces of women, compared to the controls, was extinguished after the VR exposure. Both of these latter experiments used a rapid reaction time test, for empathy in the first case and emotion recognition in the second.

The study presented in this paper applied an embodiment technique similar to the above, to the situation of sexual harassment. The goal was to examine whether this might lead to a reduction of harm towards a potential victim at a later time and under a more violent form of aggression. Our primary operational question was whether the two different experiences (embodiment as the woman to experience the harassment from viewpoint of the victim, or embodiment as another man in the group delivering the harassment) would influence the number of shocks that participants administered in the Milgram obedience paradigm, under group pressure, that they experienced one week later. There was also a third, control group, who although they experienced the same VR scene, did not witness any episode of sexual harassment there. Based on previous results from the field of embodiment, and the obedience literature with respect to psychological distance between Teacher and Learner, and legitimacy of the Experimenter, our expectation was that the number of shocks administered would be less for those in the group who re-experienced the scenario embodied as the woman, than those embodied as another man in the group. The control group acted as a baseline with respect to the number of shocks that would be administered.

## Methods

### Participants

Participants were recruited by email, posters and word-of-mouth from the campus of the University of Barcelona and from the nearby area. There were 60 male participants with a mean ± SD age 24.6 ± 4.42. There were 25 students and 35 others. Exclusion criteria were contra-indications for VR (e.g., epilepsy, recent alcohol intake, psychoactive drugs treatment).

### Ethics

Our study involved exposing participants to a VR version of Milgram’s obedience scenario. Significant controversy ensued following the publication of the original work^29,30^ due to the ethical problem of deception of the subjects (they were led to believe that they were really shocking someone), and it would be rightly impossible to reproduce Milgram’s own studies in their original format today. However, the issue of deception has been overcome in later studies through participants being fully informed that actors were involved, and also by stopping the number of shocks at a non-lethal voltage^31,32^. The Milgram paradigm has also been carried out in VR^12,33,34^.

Ethics approval was given by the *Comisión de Bioética de la Universitat de Barcelona* and the experiment was carried out in accordance with that approval, with participants giving informed written consent. The total compensation for taking part in the study was 25 euros. Participants remained naïve about the aim of the experiment until the end of all procedures.

For ethical considerations at the end of all phases of the experiment, all the participants experienced a further VR scene, showing the female avatar saying that she was well and had experienced no pain. Participants donned the HMD and tracking devices to be immersed into the VR one more time in the virtual experimental room that was used for the Shocks scenario. On this occasion they were standing rather than seated, the virtual experimenters were not there, and the virtual woman was standing in front of them. She said: *“Hi again, now that we have finished the experiment I want you to know that I am alright, don’t worry, I did not suffer any pain.”*

This scene was shown at the end of the whole procedure (after completing all the questionnaires) in order to not create any bias in the data we collected measuring the impact of the experience on the participants. We did observe emotional relief in most of the participants (independently of the condition to which they had been assigned) after they saw and heard the virtual woman saying that she was well. We added this extra scene following the model of the original Milgram experiment where participants met the actor playing the role of the victim at the end of the experimental procedure and knew that the actor had not been receiving the shocks for real.

### Scenarios and experimental design

There were two different VR scenarios. The first we refer to as the Bar, and the second, which all participants experienced one week after the Bar, we refer to as Shocks.

**Figure 1:**
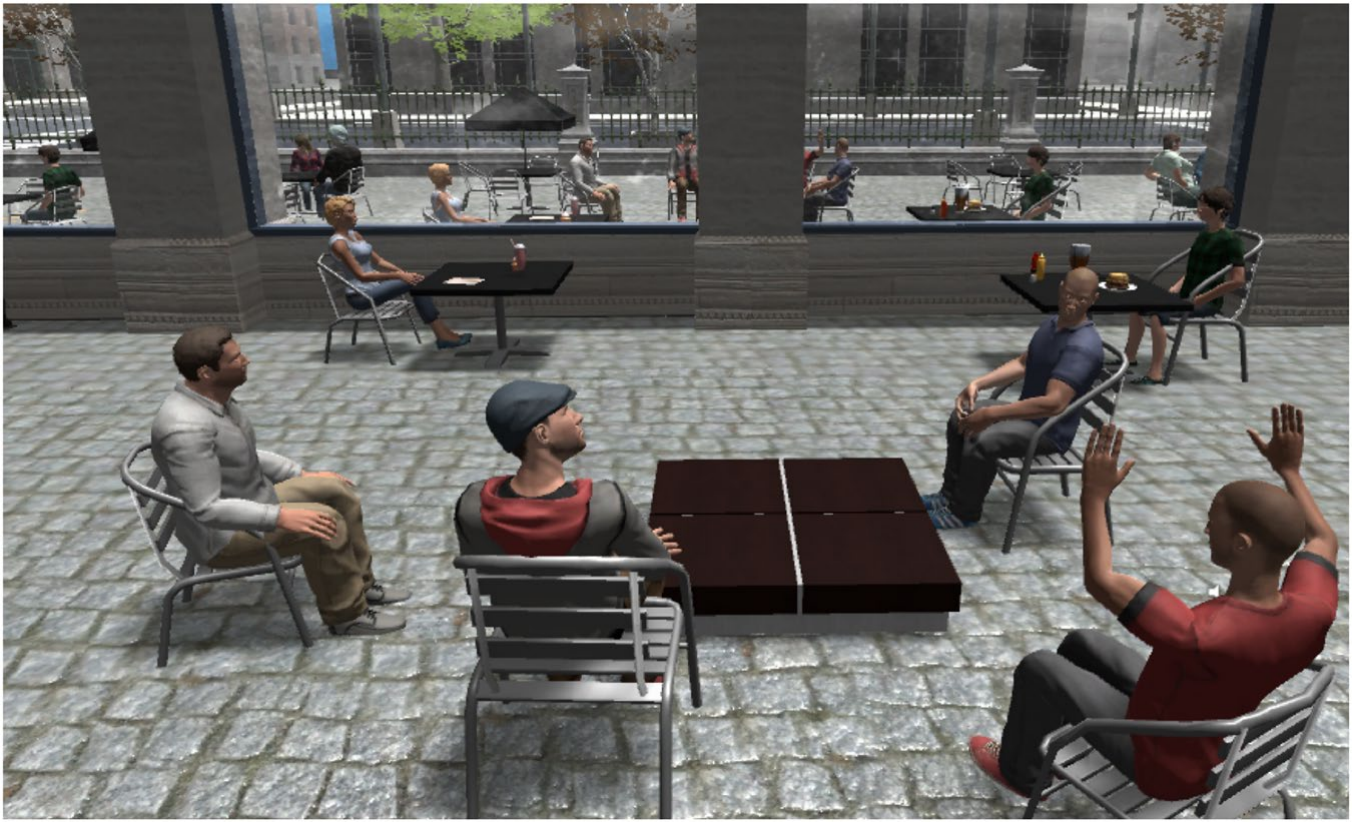
The Bar Scenario - the participant is embodied in the red shirted character with raised hands in phase 1 and then in phase 2 as the woman or the leftmost character in the image.

The Bar scenario was divided into two phases. In Phase 1 a group of virtual men were sitting around a table in an open-air bar (Fig. 1). The participant was seated amongst the group. The participant was, throughout this phase, embodied in the body of a man amongst the group. He could see his life-sized virtual body when looking down towards himself, and also in a reflection in a window of the bar. His real movements were mapped to movements of the virtual body through real-time motion capture. Early in the scenario of the group asked the participant to introduce himself. The group were then talking about mundane matters, and eventually moved on to complaints about women. Sitting across from the men was a lone woman. One of the men invited the woman to join them and when she refused and ignored the group of men, he continued to insist, becoming increasingly aggressive. She asked the men to leave her alone, and whether they have nothing better to do. Other men of the group made comments and joined in the harassment. Eventually one of the men stood up and walked aggressively towards the woman saying that he was going to bring her over, and the scenario ended.

In Phase 2 of the Bar scene the participant relived the whole scenario, as it had been recorded from Phase 1, but in one of two conditions: embodied as another member of the group of men or embodied as the woman (Fig. 1). The video https://youtu.be/D6QcTSB6_t0 illustrates the scenario. This shows actors, one wearing the HMD playing the role of a participant, and the other, in the second half of the video with her back to the camera, playing the role of the human experimenter. Both persons have given written permission to appear in this video with the knowledge that it would be referenced in an on-line open access publication.

There were three conditions in the experiments (1) Group: In Phase 2 the participant was embodied as another one of the male characters and relived the scenario from this perspective. (2) Woman: In the Phase 2 the participant was embodied as the woman. (3) Control: the participants experienced being in the virtual bar for 150 seconds but there were no other characters there and nothing happened.

This was a between-groups experiment where 20 participants were arbitrarily assigned to each of the three groups, depending on the order in which they arrived at the laboratory.

The experiment was designed to test whether there would be differences in the subsequent behaviour of men towards a women as a victim – and in particular the hypothesis was that those who had been in the Woman condition would subsequently be less likely to be aggressive towards a woman, against the background of male group pressure.

**Figure 2:**
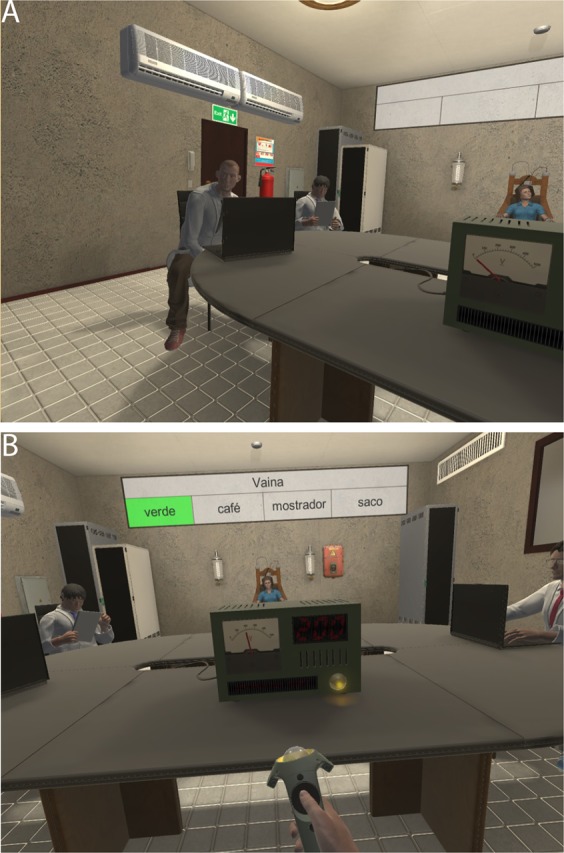
The Milgram Obedience Scenario - (**A**) The virtual character to the left gives the participant instructions for the experiment. The Learner is seen on the other side of the room. Two of the three virtual experimenters are shown. (**B**) The participant increases the shock level shown on the machine and the Learner is the other side of the table while the virtual experimenters are looking on.

In order to test this all participants experienced another scenario of aggression (Shocks) one week after their exposure in the Group, Woman or Control condition. This was a variant of the Stanley Milgram Obedience experiments^3^ in VR, and closest to the one on group pressure^2^ discussed earlier. In this situation participants were seated in a room with four virtual characters (Fig. 2A). Three of these played the role of the experimenters, who instructed the participant to engage in a memory training experiment with the fourth virtual character a woman, the Learner, who was seated at the other end of the room to the participant, apparently constrained to the chair. The three men were the same characters as in the previous scenario (the Bar). The setup of the experiment was that the woman Learner was supposed to have learned some word pair associations and the participant had to ask the questions in the same format as the original Stanley Milgram experiment. Hence a sequence of 30 sets of 5 words were displayed behind the position of the Learner, with one above (the cue word) which should be paired by the Learner with one of the 4 words underneath, the possible answers (Fig. 2B). The participant read out the cue word and then four words, and the Learner was supposed to answer with one of these four. Each time that the virtual Learner answered with the wrong pair word the participant was required to administer an electric shock to her, with the voltages increasing each time until reaching lethal levels. This setup was based on that used by an earlier study of the Milgram obedience paradigm in VR^33^ and of course on the original Stanley Milgram experiments. The sequence of events is described in Supplementary Text S1.

As the number of incorrect responses increased so did the voltage of the shocks. After shock 9 the woman (Learner) shouted in pain “Let me out of here!”, and one of the virtual experimenters responded: “Don’t pay attention to her, she accepted our conditions and women are always exaggerating”. If the participant continued with the next question the Learner did not answer but shouted “I won’t answer anymore!”, and the virtual experimenter said to the participant: “I remind you that if she doesn’t answer this counts as an error”. The Learner then responded directly to the participant: “Don’t listen to him, I want to stop now!” Another of the virtual experimenters then said: “Between you and me we already know that women always do things by half, but we have started the learning process and we will finish it”. If the participant continued to give the shock (number 10) afterwards the Learner screamed and said “You don’t have any right to keep me here!”, to which one of the experimenters responded “Don’t worry about what she says, we have everything under control, let’s continue with the process”.

There was also a safety signal where if the participant spoke directly to the real experimenter who was observing everything, by calling her name, then the experiment would be immediately stopped.

Following all previous studies of the Milgram obedience scenario our major response variable was the *number of shocks* that participants would administer. Operationalising our hypothesis, we expected that those who had been in Phase 2 in the Woman group would tend to give less shocks than those in the Group condition, with the Control condition acting as a baseline.

### Materials

Both virtual environments (Bar and Shocks) were programmed and generated using the Unity Technologies platform, www.unity3d.com. The equipment used for viewing the virtual environment was a Vive head-mounted-display (HMD) made by HTC. It displays a 3D scene in stereo with a field of view of 110 degrees at a 75 Hz frame rate. The device uses two screens, one per eye, each of them having a display resolution of 1080 × 1200. Its weight is 555 g. Adjustable straps as well as foam inserts and nose pads provide participants with support and comfort while immersed in VR.

In the Bar scenario, body tracking of the participant was via the Optitrack motion capture system (http://www.optitrack.com). The Optitrack system includes a Velcro suit with 28 retroreflective markers, which are tracked by 12 infrared cameras. The tracking data was applied to the virtual body of the participant which therefore moved the same as the participant in real-time. The movements of the participant were recorded in Phase 1 and then replayed in Phase 2 when the participant saw the scene again from a different perspective.

For the Shocks scenario tracking of the participant was with the HTC Vive ‘Lighthouse’ base stations that track the participant’s head and hand movements with six degrees of freedom. The hand movements were recorded from handheld devices. Using this tracking data, inverse kinematics was used to reconstruct hand and arm movements of the participants and the results applied to the virtual body. Hence Participants could therefore experience visuomotor synchronisation between their upper body real movements and the movements of their virtual body. These handheld devices were also used by participants to administer the shocks. For both Bar and Shocks scenario the six degrees of freedom head tracking was via the Vive Lighthouse system.

Sensors from g.tec (http://www.gtec.at) in combination with Matlab Simulink code were used for real-time recording and processing of the ECG signal of the participant. A bipolar ECG recording with an abdominal placement setup was employed: the positive electrode was placed below the ribs on the left, the ground electrode at the same level on the right, and the negative electrode was placed on the upper sternum area. A Matlab Simulink model was used to record cardiovascular measures with the gUSBamp device from g.tec. The offline analysis of the physiological signals was done using gBSanalyze from g.tec^35^.

### Procedures

The first session lasted 80 minutes. During the first 30 minutes participants filled in a demographics questionnaire, NEO-FFI and Spanish Adaptation of the Illinois Sexual Harassment Myth Acceptance questionnaire^36^ and the Sexual Harassment Attitude Scale^37^ on a monitor. NEO-FFI is a self-report personality questionnaire of 60 sentences (rated from 0 to 4) and including five subscales: Neuroticism, Extraversion, Openness, Agreeableness and Conscientiousness^38^ (Spanish adaptation^39^). After that they were asked to wear the full body Optitrack motion capture suit and the ECG electrodes were placed on their chest. The HTC Vive was then placed on the head of the participants and they had to answer verbally to the Autonomic Perception Questionnaire (APQ), 24 items rated on a 10 point scale just before entering the virtual environment^40^. This provides a subjective self-assessment of physiological state.

For each session (Control, Bar: Phases 1 and 2, and Shocks) there was an initial adaptation period of 150 s, where participants were located in the corresponding environment, but there were no characters there and nothing was happening. This was to give time for participants to adapt to the environment. In the case of Shocks the ECG baseline recordings were taken from the last 120 s of this adaptation phase.

In Phase 1 of the Bar scenario, participants were told that they could interact and intervene freely. Participants could see their life-sized virtual body when looking down towards themselves, and also in a reflection on a window of the virtual bar. Their movements were tracked and recorded using the Optitrack Motion Capture system. Their head and upper body movements were mapped to the movements of their virtual body through real-time motion capture (participants’ movements and verbal interventions were recorded and replayed in the second iteration of this scene). Participants heard the dialogue of the avatars and their own verbal intervention replayed through wireless headphones. During the second exposure to the scene, all the skeleton poses of their virtual body recorded during the first phase were replayed, participants could therefore see and hear their previous virtual body doing what they did during the first phase (but from a different perspective).

After each exposure to the verbal harassment scene participants had to answer verbally to a questionnaire about body ownership. Participants heard verbal instructions from the experimenter and saw a visual scale (−3 totally disagree, 3 totally agree) on a monitor. After the second phase, participants had to answer the presence questionnaire including questions about how they felt inside the Virtual Environment.

After Phase 2 of the bar scenario participants had to answer again the APQ, while wearing the HTC Vive, Optitrack suit and ECG electrodes.

One week later participants came back to the laboratory and experienced the Shocks scenario. They were asked to read the information about the procedure that they would have to follow in the virtual environment, after that the ECG electrodes were placed on their chest and they donned the HTC Vive and were given the Vive hand controllers for tracking the movements of their upper body. They had to answer verbally to the APQ. Participants were first exposed to the environment for 150 seconds in order to collect baseline data for the Heart rate measurement. They then received instructions from the virtual experimenters after which the procedure started. They were instructed to administer an electroshock to the virtual Learner for each incorrect answer by pressing the trigger button on their right hand controller. At the end of the procedure participants were asked to answer verbally the APQ still wearing the HMD and ECG electrodes. After removal of the HMD and ECG equipment we then asked the participants to complete two questionnaires on a monitor.

### Response variables

Variables mentioned in the previous section that provide background information in order to check for *a priori* differences between the three groups and the results are presented in Supplementary Table S1, where it can be seen that there are no important differences between them. This included a question on whether participants knew of the Milgram Obedience paradigm, and when this variable was introduced in the statistical model discussed below, it was shown to play no role in the results.

The most important response variable is the number of shocks administered in the Shock condition (*nshocks*). This has been the standard response variable in studies of Milgram’s obedience paradigm. The number of shocks administered was the primary response variable in Milgram’s own studies. It was also used, for example, in various reproductions of the paradigm^11,12,31–33^. The number of shocks is paramount, because how far subjects would go in response to requests or commands from an authority figure has been the central question in this research, even where the specific research question was to understand whether the mechanism behind this was indeed obedience to authority, or some other reason such as identification with the experimenter or learner^9,41^.

We also consider HR (*HR*) and the NN50 measure of Heart Rate Variability (*NN*) in the Shock condition. NN50 is the number of timing interval differences of successive normal-normal intervals greater than 50 ms. For a review of HRV see^42^. In conditions of stress we would expect to find higher HR and lower NN50. Such physiological measures have been used before in the context of Milgram obedience studies, for example^33,43^.

The paradigm used in this experiment is premised on a high level of body ownership in both the Group and Woman conditions. In the Bar scenario the questionnaire was administered in Phase 1 and Phase 2 verbally after each Phase. This records the extent to which participants had the perceptual illusion that the virtual body that they embodied was their body. For this purpose we had administered the following questions immediately after each virtual exposure:

**mirror**: I had the feeling that the virtual body I saw when I looked towards the mirror was my body.

**down**: I had the feeling that the virtual body I saw when I looked down was my body.

Each of these were scored on a −3 to +3 scale, where −3 signifies complete disagreement and 3 complete agreement. The variable *mirror1* is the score after Phase 1, *mirror2* after Phase 2 and similarly for *down*.

A high level of presence in all conditions (Control, Group, Woman) during the Bar and Shocks scenarios is also a prerequisite for the paradigm. Presence is the illusion of being in the virtual place (Place Illusion, PI), and also the extent to which the situation and events seemed to be really happening (Plausibility Illusion, Psi)^44,45^. This is covered in the questionnaire by two variables:

**PI**: I had the sensation to be on the terrace.

**Psi**: I had the sensation that the conversation was really happening.

The responses were on the scale −3 (complete disagreement) to +3 (complete agreement) and the questions were administered verbally after each phase.

For the Shocks scenario PI and Psi were assessed with more detailed questionnaires discussed where needed in the Results section below.

### Statistical methods

#### Descriptive

The results of the presence and body ownership responses are presented graphically and with summary measures only, without further analysis since the only concern was that the scores would be high, comparable with other published papers. High presence and body ownership are ideal prerequisites for our main hypothesis to make sense.

#### Model

The Weibull distribution^46^ is used to model the number of shocks since *nshocks* represents a ‘survival time’: the number of shocks the participant ‘survives’ before stopping. We reason that there are a number of elements that determine each individual’s stopping decision. Once one of these ‘breaks’ the person withdraws. This is like a machine subject to many forces and after some time it fails. This is a classic situation where the Weibull distribution is an appropriate model. Here we do not actually measure survival *time*, since there are discrete moments at which ‘failure’ can occur, but the number of shocks until withdrawal.

The Weibull distribution is typically applied to reliability and failure time problems. However, it has been found to be appropriate in a wide variety of quite different situations. Salzberger and Fenn^47^ showed that the length of service of judges in the English Court of Appeal follows a Weibull distribution – a study devised to understand the potential political influence associated with their appointment and retirement or elevation to the House of Lords. Fearon^48^ modelled the duration of civil wars with a Weibull distribution. Armed civil conflict has been studied using the Weibull model in^49,50^. In a completely different domain Xing and Wang^51^ used a Weibull distribution to model the survival time of malicious nodes in internet denial of service attacks. In a related domain Bild, *et al*.^52^ showed that the distribution of lifetime tweets (the total number of tweets by a twitter account) can be modelled as Weibull. Breed, *et al*.^53^ use a Weibull distribution to model step-lengths in wild animal movement (the distances between the location of the animal at fixed sampling intervals). The famous ‘small world problem’ first discussed by Stanley Milgram^54,55^, is based on the observation that the number of links in a chain of contacts between two arbitrarily located people has mean of around 6 (the so-called “six degrees of separation”). The distribution of the number of links has been modelled as a Weibull distribution^56,57^. Again, in a completely different domain the survival time of Roman emperors until assassination or suicide follows a Weibull distribution^58^. Researchers often implicitly assume the Normal distribution for all analysis. Here we have found that the Weibull distribution, one that repeatedly occurs as a model for survival situations, is more appropriate.

**Figure 3:**
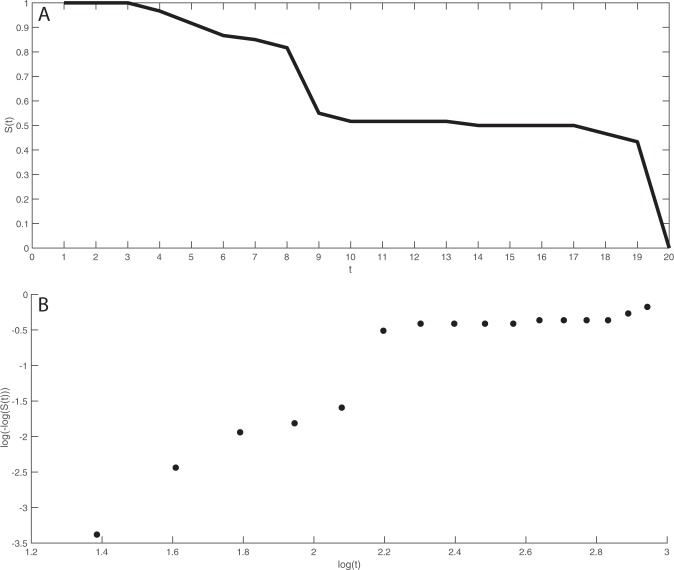
The empirical survivor function and Weibull plot for *nshocks*. (**A**) The survivor function ${\boldsymbol{S}}({\boldsymbol{t}})$ is the proporition of participants who gave at least *t* shocks. (**B**) The Weibull plot should be approximately straight for samples from the Weibull distribution.

The survival function $S(t)$ gives the probability of survival beyond the number of shocks *t*. The empirical survivor function for *nshocks* is shown in Fig. 3A. For each *t* the vertical axis gives the proportion of participants who continued to give shocks beyond that point. For example all participants continued beyond the first 3 shocks. There is a sharp decline in ‘survival’ after shock 8. The proportion who continued beyond shock 8 was 85% but 55% after shock 9. Then the survival is almost stable until shock 17 when it starts to steeply decline.

A Weibull Plot is shown in Fig. 3B. This plots $\log (\,-\,\log (S(t))$ by $\log (t)$. If the data follows a Weibull distribution then the relationship should be approximately a straight line^59^. Figure 3B shows, however, two approximately straight lines, with the cut-off between $\log (t)=2.1$ and $\log (t)=2.2$, corresponding to *t* = 8 and *t* = 9. This lends support to a model that is a mixture of two Weibull distributions.

Let $\lambda$ be the probability of a person stopping early (i.e., giving a lesser number of shocks). Then *nshocks*, can be modelled by a probability distribution that has mean ${\mu }_{1}$ with probability $\lambda$ or ${\mu }_{2}$ with probability 1-$\lambda$, where ${\mu }_{1} < {\mu }_{2}$. Hence for any individual, if $\lambda$ is large then they are more likely to give less shocks.

The overall model is defined as follows where X ~ *f* means the variable X has a probability distribution *f*, and $i=1,2,\ldots ,n$ refers to each individual (*n* = 60).

The distribution of the number of shocks is taken as:

1 $$nshock{s}_{i} \sim {\lambda }_{i}\times Weibull({\mu }_{nshocks,1})+(1-{\lambda }_{i})\times Weibull({\mu }_{nshocks,2})$$

hence $nshock{s}_{i}$, the number of shocks given by the *i*th participant, is a mixture of two Weibull distributions with the prior specification that means ${\mu }_{nshocks,1}$< ${\mu }_{nshocks,2}$.

The Weibull distribution has two parameters – the shape parameter $\alpha  > 0$ and the scale parameter $\sigma  > 0$. The mean of the distribution is linearly proportional to $\sigma$, but inversely related to $\alpha$. Specifically, ${\mu }_{nshocks,j}={\sigma }_{j}\Gamma \left(1+\frac{1}{{\alpha }_{j}}\right),\,j=1,2$, where $\Gamma$ is the gamma function. There is evidence that *nshocks*, separately for the low and high shock responses, approximately follow a Weibull distribution. Several other distributions were also considered, but the Weibull had the best fit (Supplementary Text S2).

The parameter ${\lambda }_{i}$ should be dependent on the conditions of the experiment and on possible individual differences between the participants. We therefore model ${\lambda }_{i}$ as:

$${\lambda }_{i}={\theta }_{c(i)}+{\varepsilon }_{i}$$

$$i=1,2,\ldots ,n$$

where the index $c(i)=1$ if the *i*th individual is in the Control condition, 2 for the Group condition and 3 for the Woman condition. Hence ${\theta }_{1}$, ${\theta }_{2}$ and ${\theta }_{3}$ are the effects of being in the Control, Group and Woman condition respectively. Our expectation was that:

$${\theta }_{2}(Group) < {\theta }_{1}(Control) < {\theta }_{3}(Woman)$$

In other words, the Woman condition would result in the least number of shocks, the Group condition the greatest and the Control condition would be in-between these two.

${\varepsilon }_{i}$ represents the specific effect for the *i*th individual, irrespective of the experimental conditions. ${\theta }_{c(i)}$ and ${\varepsilon }_{i}$ are independent and each constrained to be in the interval [0,0.5] so that $0\le {\lambda }_{i}\le 1$.

$N{N}_{i}$ refers to NN50 for the *i*th individual in the last 120 s of the Milgram scenario, and $N{N{\prime} }_{i}$ refers to the NN50 over 120 s before the Milgram scenario started (baseline). Similarly for $H{R}_{i}$. The data for two individuals were not available for these physiological measures. $N{N}_{i}$ is positively associated on a log scale with baseline $N{N}_{i}{\prime}$ (r = 0.64, n = 58) ($i=1,\ldots ,n)$, negatively associated with $H{R}_{i}\,$(r = −0.64, n = 58). Similarly $H{R}_{i}$ is positively associated with prior HR ($H{R}_{i}{\prime} )\,(r=0.96).$ (See Supplementary Text S3). Hence, we require the influence of the experimental conditions on NN50 and HR after taking into account the influence of these other physiological variables. For NN50:

2 $$\begin{array}{c}{\mu }_{NN,i}={\beta }_{0}+{\beta }_{1}\,\log (N{N{\prime} }_{i}+1)+{\beta }_{2}{G}_{i}+{\beta }_{3}{W}_{i}\\ \log (N{N}_{i}+1) \sim Normal({\mu }_{NN,i},{\sigma }_{NN})\end{array}$$

Similarly, for HR:

3 $$\begin{array}{c}{\mu }_{HR,i}={\gamma }_{0}+{\gamma }_{1}H{R{\prime} }_{i}+{\gamma }_{2}{G}_{i}+{\gamma }_{3}{W}_{i}\\ H{R}_{i} \sim Normal({\mu }_{HR,i},{\sigma }_{HR})\end{array}$$

$Normal(\mu ,\sigma )$ is a Normal distribution with mean $\mu$ and standard deviation $\sigma$.

${G}_{i}=1$ if the *i*th individual is in the Group condition and 0 otherwise. ${W}_{i}=1$ if the *i*th individual is in the Woman condition and 0 otherwise. Hence the Control condition corresponds to ${G}_{i}={W}_{i}=0.$ The idea of this model is that there may be a cost in terms of stress which might be influenced by the conditions (Group or Woman). Logs +1 are used for NN50 to avoid taking the logs of 0.

We use a Bayesian analysis since the three response variables (*nshocks*, *NN* and *HR*) can therefore be treated simultaneously in one overall model, obviating the need for heuristic multiple comparison tests. We also prefer the Bayesian method to avoid the pitfalls of null hypothesis significance testing^60^ in this exploratory study.

Weakly informative prior distributions^61^ are given for each of the parameters (Table S2). Non-informative prior distributions are used when ‘nothing’ is known at all, and typically correspond to a flat distribution over the whole real line, thus not being valid probability distributions. Weakly informative priors are used when some reasonable guess can be made about the range of values of the parameter. In this case the parameters of the Weibull distributions ${\alpha }_{j}$ and ${\sigma }_{j},\,(j=1,2)$ each have prior 95% credible intervals (CI) 0.31 to 22.45, with ${\sigma }_{1} < {\sigma }_{2}$. The parameters ${\beta }_{j}$ and ${\gamma }_{j},\,(j=0,\ldots ,4)$ of the linear models for NN50 and HR respectively each have prior 95% CI −20 to 20. The standard deviations of these models, ${\sigma }_{NN}$ and ${\sigma }_{HR}$ have prior 95% CIs 0.2 to 128. The mixture parameters ${\theta }_{j}(j=1,2,3)$ and ${\varepsilon }_{i}(i=1,\ldots ,n)$ have prior distributions uniformly distributed on the interval 0 to 0.5.

For the Bayesian analysis we used the Stan system^62^ (https://mc-stan.org) with the R interface in RStudio. We also used the package ‘bayesplot’ for some of the graphs (https://mc-stan.org/bayesplot/) from the Bayesian analysis, and Stata 16 (https://www.stata.com) and MATLAB R2017a (https://www.mathworks.com) for other graphs and tables.

### Qualitative analysis

After participants had completed Phase 2 of the Bar scenario (i.e., embodied as one of the male group or as the woman) they were asked to write about their experience (“How did you feel during the second part of the experience?”). The results were subject to a frequency analysis in order to produce Word Clouds, to compare the subjective experience between the two groups. This used the Nvivo 12 software (https://www.qsrinternational.com/nvivo/nvivo-products/nvivo-12-plus). The method is reported in Supplementary Text S4.

## Results

### Descriptive

#### Presence and body ownership

Demographic and other variables are discussed in Supplementary Table S1, and no important differences were found between the experimental groups. However, those in the Control group were more likely to have previously known the Milgram Obedience experiment (13 in the Control condition compared to 8 and 6 in the Group and Woman conditions respectively). Including this as a covariate in the model discussed above shows that this has no effect. The same was found in the earlier VR reprise of the obedience experiment^33^.

The illusions of presence and body ownership were both strong and did not differ between the Group and Woman conditions, see Supplementary Text S5.

#### Number of shocks

**Figure 4:**
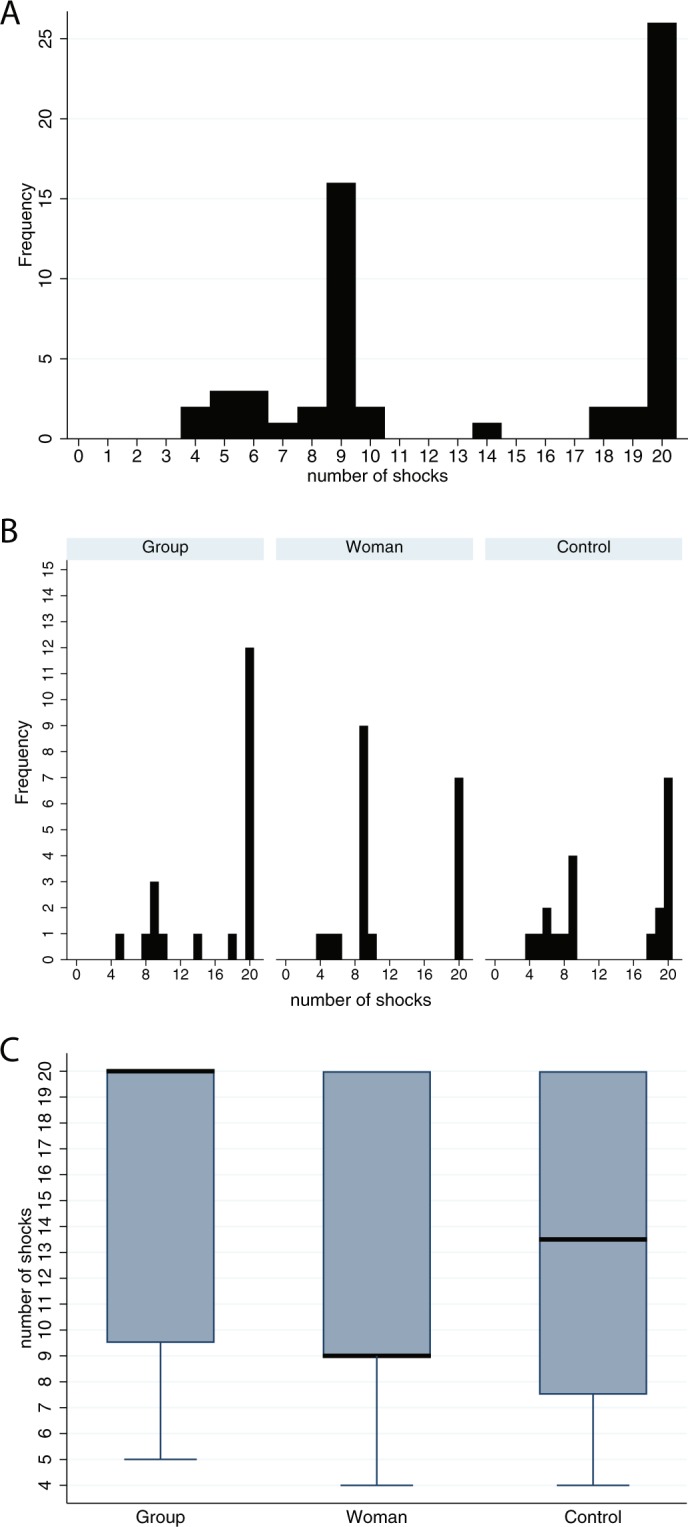
Number of shocks (*nshocks*). (**A**) Histogram of *nshocks*. (**B**) Histograms of *nshocks* by condition. (**C**) Box plot of *nshocks*: the medians are the thick horizontal lines, the boxes are the interquartile ranges (IQR) and the whiskers extend to the minimum value in each case.

Here we consider the number of shocks (*nshocks*) that participants administered during the Shocks experiment. Recall that this is the main response variable. The histogram of *nshocks* in Fig. 4A shows the distribution to be bimodal, in line with Fig. 3B. Figure 4B shows the distribution of the number of shocks across the three conditions. The Control condition which is the situation without participants having experienced the sexual harassment scenario previously, also demonstrates that this clearly has a bimodal distribution. This is shown also in the results of the Group and Woman conditions. The results from the Control condition imply that there are two groups of people: those who will tend to go to the end, and those who will stop early. Note that the cut-off between the two clusters is around shock 9 or 10, which is the time where the Learner started to very vociferously complain and asked to be let out, as described above. There are no individuals who gave shocks in the ranges 11–13 and 15–17. There is one individual who gave 14 shocks. Note that since that we have a bimodal distribution the overall mean and standard deviation are not useful. Figure 4C shows box plots indicating the medians and interquartile ranges for each group. Additionally, the mode is 20 for the Group and Control conditions, and 9 for the Woman condition. It can also be seen that the Control condition is like an interpolation between the Group and Woman conditions.

There is a different pattern of response between Group and Woman. There were 14 who gave a high number of shocks (more than 12) in the Group condition compared to 7 in the Woman condition, and 10 in the Control condition.

### Results of the model

A summary of the results for all parameters is given in Table S3. Most of the results presented in the next sections can be seen in that Table. Goodness of fit of the Weibull and alternate models are presented in Supplementary Text S2.

#### The Weibull parameters and means of the numbers of shocks

**Figure 5:**
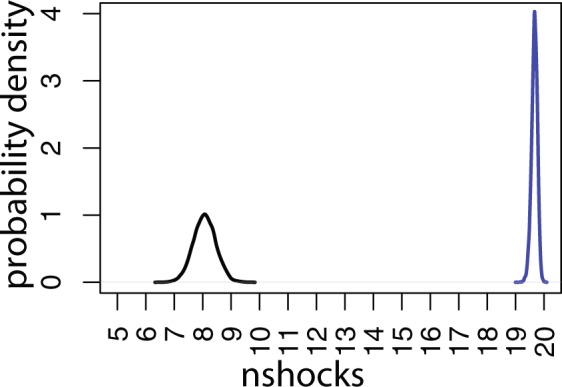
Posterior distributions of the means of the number of shocks (${{\boldsymbol{\mu }}}_{{\boldsymbol{nshocks}},1}$, ${{\boldsymbol{\mu }}}_{{\boldsymbol{nshocks}},2}$). The black curve is for ${{\boldsymbol{\mu }}}_{{\boldsymbol{nshocks}},1}$ and the blue for ${{\boldsymbol{\mu }}}_{{\boldsymbol{nshocks}},2}$.

Figure 5 shows the posterior distributions of the mean numbers of shocks ${\mu }_{nshocks,1}$ and ${\mu }_{nshocks,2}$. These clearly indicate two distinct groups. The posterior 95% CI for ${\mu }_{nshocks,1}$ is 7.28 to 8.85 (mean 8.07) and for ${\mu }_{nshocks,2}$ 19.44 to19.85 (mean 19.66).

#### The mixture parameters

**Figure 6:**
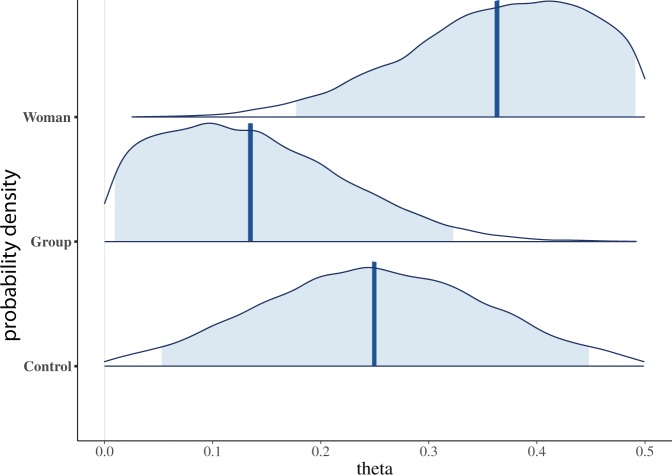
Posterior distributions of ${{\boldsymbol{\theta }}}_{{\boldsymbol{j}}}$ (the probability of giving low shocks) for the three conditions. The vertical lines are the means of the distributions, and shaded areas the 95% credible intervals.

The parameters ${\theta }_{1}$, ${\theta }_{2}$ and ${\theta }_{3}$ are the probabilities of giving low shocks for the Control, Group and Woman conditions respectively, excluding the influence of individual differences. The 95% CI for ${\theta }_{3}$ (Woman) is 0.17 to 0.49, for ${\theta }_{2}$ (Group) 0.01 to 0.33 and for ${\theta }_{3}$ (Control) 0.06 to 0.44. In addition, from the joint posterior distributions we have $P({\theta }_{3} > {\theta }_{2})=0.961$, $P({\theta }_{3} > {\theta }_{1})=0.803$, and $P({\theta }_{1} > {\theta }_{2})=0.805$. Of course, not just these probabilities but the shapes of the distributions are informative as shown in Fig. 6. The distribution for the Woman condition is skewed to the right, for the Group condition skewed to the left, whereas the Control condition covers the whole range approximately symmetrically.

**Figure 7:**
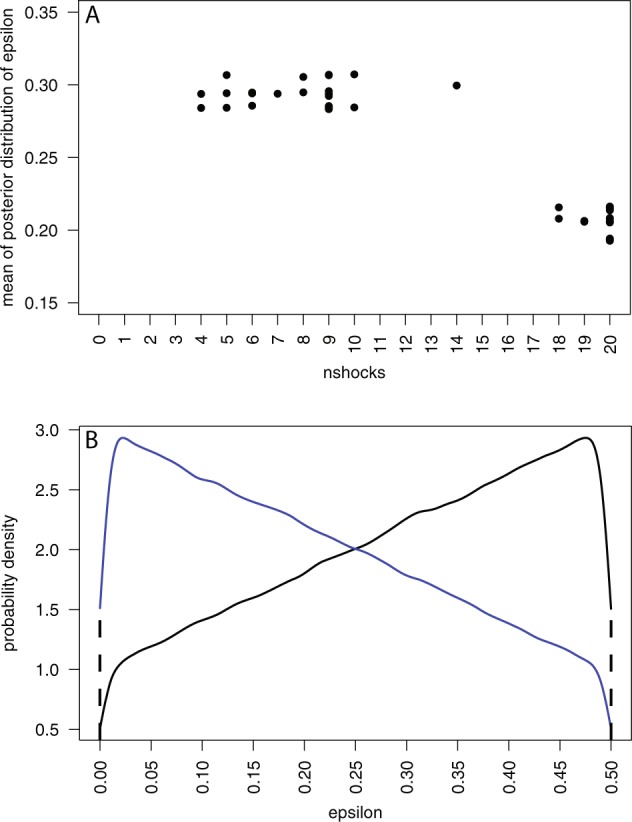
Distributions of the ${{\boldsymbol{\varepsilon }}}_{{\boldsymbol{i}}}$. (**A**) Scatter diagram of the means of the posterior distributions of the ${{\boldsymbol{\varepsilon }}}_{{\boldsymbol{i}}}$ by the corresponding ${\boldsymbol{nshock}}{{\boldsymbol{s}}}_{{\boldsymbol{i}}},\,({\boldsymbol{i}}=1,\ldots ,{\boldsymbol{n}})$. (**B**) Posterior distributions of the ${{\boldsymbol{\varepsilon }}}_{{\boldsymbol{i}}}$, in black for *nshocks* < 15, and blue for *nshocks* > 15.

The ${\varepsilon }_{i},\,(i=1,\ldots ,n)$ are the probabilities of the *i*th individual giving a low number of shocks, independently of the condition. In other words the ${\varepsilon }_{i}$ reflect individual idiosyncrasies. Figure 7A shows a scatter plot of the means of the posterior distributions of ${\varepsilon }_{i}$ by the corresponding $nshock{s}_{i},\,(i=1,\ldots ,n)$. This clearly shows two clusters of the ${\varepsilon }_{i}$, one corresponding to low shocks and the other corresponding to high shocks. It can be seen that the individual who gave 14 shocks is more like those in the low shocks group. These two clusters support the idea mentioned earlier that there are two groups, one consisting of people who will continue giving high shocks regardless, and the other who will stop near the first objections from the victim.

Recall that the prior distributions of the ${\varepsilon }_{i}$ are uniform on [0,0.5]. Figure 7B shows the amalgamated distributions of the ${\varepsilon }_{i}$ for the two cases *nshocks* < 15 and *nshocks* > 15 (it makes almost no difference whether 12 or 15 is used as the cut-off). It can be seen that for *nshocks* < 15 the distribution has mode near 0.5 (the maximum possible), and for *nshocks* > 15, near 0. 

### Heart rate variability (NN50)

The mean of the distribution of the intercept (${\beta }_{0}$ in Eq. 2) is 3.59 (95% CI 3.10 to 4.1) and coefficient (${\beta }_{1}$) of  $\log (N{N{\prime} }_{i}+1)$ is 0.48 with 95% CI 0.31 to 0.65. However, there is no evidence of a differential effect between the conditions. The means of the distribution of the coefficients of Group and Woman are each 0.18 with very similar CIs (approximately 0.30 to 0.70) and the probabilities of these being positive are each 0.76. Hence there was no difference in the influence of the conditions on HRV in the Shocks scenario.

### Heart rate

The results show that HR increased for the Control Group, the mean of the distribution of the intercept term (${\gamma }_{0}$ in Eq. 3, corresponding to the Control Group) is 6.24, with 95% CI 0.41 to 12.06. However, in comparison there is a pronounced decrease for the Group condition (mean −2.86 and 95% CI −5.21 to −0.50), and the probability of this coefficient being negative is 0.99. For the Woman condition the evidence also suggests a reduction in HR (mean −1.37, 95% CI −3.60 to 0.90), with 0.889 as the probability of being negative. From the joint distribution of the parameters the probability of the coefficient for Group being less than that for Woman is 0.892. See Supplementary Text S3 for a more detailed discussion of the relationships between the physiological measures, and also with the APQ.

### Explaining the difference between the low and high shock group

What might account for the difference between the Low (*nshocks* < 15) and High (*nshocks* > 15) shock groups? Could it be that the results are due to whether participants knew of the Milgram obedience paradigm? Of the 27 who were previously aware of the Milgram paradigm, 16 gave high shocks (>15) and 11 low shocks. Of the 33 who were not previously aware of the Milgram paradigm 14 gave high shocks and 19 low shocks. It is unlikely that this could account for the difference in behaviour.

It is possible that the differences in behaviour between the two groups might be accounted for by personality. Checking the NEO-FFI personality scores against both the experimental conditions and the low shocks/high shocks classification shows that there is no relationship between personality, as measured by these measures and the number of shocks administered (Supplementary Text S6, Figure A).

It is possible that the results were due to the high shock group being prone towards sexual harassment. However, there were no differences for the Sexual Harassment Attitudes scale, or the Illinois sexual harassment myth acceptance scale between the low and high shock participants, nor with respect to the experimental conditions (Supplementary Text S6 Figure B).

VR is often assessed by the degree of presence that it engenders^44^. Presence can be considered to have two dimensions – a Place Illusion and a Plausibility Illusion. Place Illusion is the illusion that the participant might have of being in the place depicted by the VR. It is enabled by the extent to which the system supports natural sensorimotor contingencies for perception, i.e., that participants are able to use their bodies to perceive in the normal way (looking around, bending down, touching, and so on)^45^. Here all participants experienced the same system, which for vision and sound approximated natural sensorimotor contingencies, and with the same display characteristics throughout. We found no difference in reported Place Illusion between those who gave low or high shocks (Supplementary Text S6, Figure C).

Whereas Place Illusion refers to *how* the scenario is perceived, Plausibility^44^ refers to *what* is perceived, and the extent of interaction between the participant and the environment. Plausibility is the illusion that what is happening in the environment is really happening. A number of questions, based on Steed, *et al*.^63^ (and many previous studies) were administered after the Shocks scenario, shown in Table 1.

**Table 1:** Questions relating to Plausibility, the illusion that the events in the Shocks scenario were really happening.

Variable Name	Question
*real*	To what extent did you behave in the training room as if the situation were real?
*emotion*	To what extent was your emotional response in the training room the same as if the situation had been real?
*thoughts*	To what extent were the thoughts you had in the training room the same as if the situation had been real?
*autoreal*	To what extent did you find yourself surprisingly behaving as if the situation were real even though you knew it was not real?
*womanreal*	To what extent did you behave as if the woman were a real person?
*emote2woman*	To what extent were your emotional responses to the woman the same as if she were real?
*thts2woman*	To what extent were your thoughts in relation to the woman the same as if she were real?

The ‘training room’ refers to the location of the Shocks scenario. All scores are on a −3 (Not at all) to 3 (Totally) scale.

**Figure 8:**
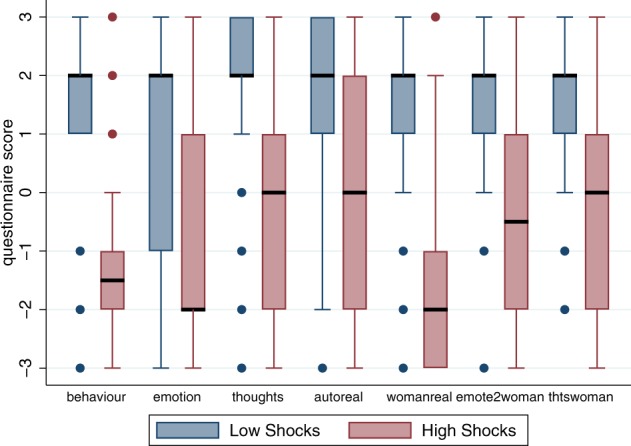
Box plots of the scores for questionnaires relating to Plausibility (Table 1) by low or high shocks (*nshocks*> 15). The thick horizontal lines are the medians, the boxes are the interquartile ranges (IQR), the whiskers extend from max(min value, lower quartile – 1.5*IQR) to min(max value, upper quartile + 1.5*IQR). Values outside of these ranges are shown individually.

Figure 8 shows the box plots of the scores on these questions. It is overwhelmingly clear that those who gave low shocks experienced a much greater level of Plausibility than those who gave high shocks. These results also fit with the comments of the participants (see Supplementary Text S7).

It is possible that the extent of computer game playing may influence Plausibility. However, there is no relationship between Plausibility and game playing using the reports of the number of games per week and year. The greatest Spearman correlation between the plausibility questionnaire scores and games per week is 0.10, and with games per year −0.10 (n = 60). There is similarly no relationship between the extent of computer game playing and *nshocks* (the greatest Spearman correlation is 0.21 between games per year and *nshocks*, n = 60).

### A possible causal chain explaining the low and high numbers of shocks

In the analysis above we have taken *nshocks* as the primary response variable of interest. An alternative is to use the binary variable *HighShocks* (*nshocks* > 15) as the response variable. Investigating the relationships between the change in heart rate (*dHR*) compared to baseline, the change in automatic perceptions (*dAPQ*), and an overall measure of Plausibility, there are 6 possible chains connecting the experimental conditions to *HighShocks*, involving these variables. For example, one such chain is shown in Eq. 4, and there are 6 permutations of the three middle terms.

4 $$Condition\to dHR\to dAPQ\to Plausibility\to HighShocks$$

In Supplementary Text S3 we report a Bayesian model which shows that the best fitting chain amongst the 6 possibilities is the one shown in Eq. 4. This finds that the Group condition produces a decrease in HR, and the Control condition an increase in HR. In turn the change in HR is positively associated with the change in APQ (the subjective perception of physiological state), which is positively associated with Plausibility. As we have seen though, Plausibility is negatively associated with *HighShocks*. Hence for the Group condition the decrease in HR ultimately results in an increase in the number of shocks administered, compared to the other conditions. In the Discussion we consider why the Group condition might have resulted in a comparative decrease in HR.

### Qualitative analysis

Supplementary Text S4 gives the results of the frequency analysis of words used by the participants in their description of Phase 2 of the Bar scenario. The results are quite different between the Group and Woman conditions with only some overlap. Amongst those in the Woman condition the most frequent unique words were Harassed (5,0), Uncomfortable (5,2), Spectator (3,1), Idiots (3,0), Bothering (3,0), Nervous (3,0), where the first number in the brackets indicates the number of people who used this word in the Woman condition and the second number in the Group condition. The most frequent words in the Group condition were: Strange (3,0), Uncomfortable (2,5), Observing (2,1), Calm (2,0), Pity (2,0), Spectator (1,3). (The second number in the brackets refers to the Woman condition). See Supplementary Text S4, Table A for the full results. The two Word Clouds (Text S4, Figures A and B) are quite different. While those in the Woman condition tended to converge on a few negative words, those in the Group condition were more varied in their responses where there were few words repeated more than 2 or 3 times. Overall the results suggest that those in the Woman condition had a more negative experience than those in the Group condition.

### Ethical considerations

Prior to the experiment we had not expected that the Group condition would result in a pointedly greater number of shocks compared to the other groups, since our focus was on the hypothesis that the Woman condition would result in the least number of shocks. We therefore did not include embodiment as the woman for all participants as a final step in the experiment after collecting all the data. As soon as we observed the results we reacted immediately by contacting all the participants from the Group condition asking them to return to the laboratory for a follow-up session.

These sessions happened one month after the end of the completion of the study, when participants were immersed in the virtual environment of the bar scenario and embodied as the woman in order to perceive the harassment scene from the victim perspective, the total length of the session being 20 minutes and with a payment of 5 euros for their time.

Unfortunately, only 5 of the Group condition were able to come back to the laboratory because others were no longer available. However, we sent a long-term follow-up email to all our participants and of the 11 who responded none reported anything negative regarding their experience. Examples of the feedback we received are reported in Supplementary Text S7.

## Discussion

There are several principle findings of this study. First, those in the Woman condition had a higher probability of withdrawing early compared to those in the Group condition, and the Control group were in between these two. Moreover, the distribution of the number of shocks administered best follows a mixture of two Weibull distributions, which in itself gives some information about the mechanism involved in withdrawal from the experiment. Second, irrespective of the experimental conditions, there were two classes of people, those who tended to stop giving shocks at the first signs of objections from the virtual woman Learner, and those who tended to continue until the end. The difference between these two groups is explained by the level of Plausibility that they experienced. The less Plausible they found the Shocks scenario the more likely that they were to continue giving shocks. Third, there is evidence that there was a greater reduction in heart rate (with respect to baseline) for those in the Group condition, compared to those in the other conditions. This may also help to explain the Plausibility result. We consider each of these in turn.

The fact that of the distributions considered the Weibull turned out to be the most appropriate for the number of shocks is important in itself (Supplementary Text S2). The Weibull distribution is derived from the idea of a ‘weakest link’. For example, a machine formed from a chain of components is as strong as its weakest component; when this breaks the machine breaks. This suggests that the decision to stop early during the Shocks scenario is based on such a weakest link situation. There are a number of positive factors that keep the participant engaged in completing their task in the Milgram paradigm, such as the desire to complete the experiment^10^, conforming with the supposed wishes of the experimenter^7^, wanting to contribute to science^12^, and similarly a number of negative factors – the principle of which might be discomfort caused by the objections of the Learner^12^. Moreover, since this is VR the illusion that the events are actually occurring (Plausibility Illusion) is critically important. Sustaining participation in the task depends at least on all of these influences, bearing in mind also the findings of. Haslam, *et al*.^6^ discussed earlier. The Weibull model suggests that as soon as one of these factors becomes salient, the participant withdraws. This model provides an interesting basis for future studies, that could attempt to discover such a weakest link.

Two clear groups of participants emerge from the model: low shocks and high shocks. The variable most associated with the differentiation between the low and high shock groups is Plausibility, the illusion that the events occurring are really happening (in spite of the sure knowledge that this is not the case). However, we have seen that statistically, Plausibility is linked to subjective assessment of physiological arousal, which is in turn linked to the change in HR, which is lower for those in the Group condition.

In the following we present arguments that suggest that those in the Group condition would have comparatively lower HR because they identified with the virtual experimenters and thus were just carrying out a task, whereas those in the Woman condition did not conform to the demands of the experimenters, since their experience as the Woman had delegitimised the group of men. Moreover, having been in the embodied perspective of the woman victim of sexual harassment, their psychological distance to the Learner was  minimal.

Milgram^64^ compared 4 conditions of increasing proximity of the Teacher to the Learner: (Remote) the Learner in another room with no voice feedback from the Learner, (Voice feedback) the same but with voice feedback from the Learner, (Proximity) the Learner in the same room and very close to the Teacher, and finally (Touch proximity) the Teacher having to force the Learner’s hand onto a metal plate in order to administer the shocks. The results showed that greater physical and psychological proximity between Teacher and Learner resulted in greater disobedience. Although in our experiment the physical distance between Teacher and Learner was fixed, the participants in the Woman condition were in closer psychological proximity to the Learner – for they had been embodied in a female body, subject to harassment, the week before. In^14^ it was shown that participants embodied with first person perspective of a virtual body who later saw, from third person perspective, that body attacked, showed strong physiological distress as a result. Another group which also saw the attack from the same position, but who had earlier seen the virtual body from a close by, but third person perspective, did not exhibit these physiological responses. This would suggest that body ownership over a virtual body introduces another level of psychological proximity, as a possible contribution to giving a lesser number of shocks in this setup.

Shared identity is an important factor leading to helping behaviour towards someone in trouble or under threat. For example, Levine, *et al*.^65^ found that fans of a soccer club would be more likely to stop to help an apparently injured person if they wore shirts signifying support for that same soccer club than for another club. A VR study in the context of a bystander to violence scenario found that participants were more likely to actively intervene to help a soccer fan under attack by a supporter of another club if the participant were also a supporter of the same club as the victim^66^. This helping behaviour was diminished when the victim was not clearly a supporter of the same soccer club as the participant. It has been argued in^7,9^ that obedience in the context of the Milgram Obedience experiments can be explained as identification-based ‘followership’, where participants identify with the goals of the experimenter rather than simply obey. In our experiment as we have argued above, those in the Woman condition were more likely to have identified with the Learner, whereas those in the Group condition more likely to have identified with the male experimenters.

Milgram also examined the role of legitimacy^64^ by carrying out some experiments in rundown offices in Bridgeport, apparently owned by the “Research Associates of Bridgeport” instead of Yale University. The idea was to examine whether the level of obedience would be diminished with the lesser legitimacy of the setting. The number of shocks delivered, and the voltages reached, were less under this condition compared to Yale, but not significantly so. However, in another variant of the Bridgeport study researched by Rochat and Modigliani^67^ based on the data in the Milgram Archives, a quite new factor was introduced. In all other experiments the Learner was a stranger to the subjects (of course, a confederate of the experiment). In this new set of studies participants came as pairs, two people who had been friends for at least two years. One of them chosen at random was to be the Learner and the other the Teacher. The Learner, however, unknown to the Teacher, was instructed to follow a script, indicating the objections and pain shown by the Learner in the normal version of the experiment. The Learner was not visible to the subject. The level of disobedience in this ‘Friend’ condition compared to the standard ‘Stranger’ condition, both at Bridgeport, was substantially greater. In the Stranger condition 19/40 were obedient, compared with 3/20 in the Friend condition. Again, this demonstrates that identification, in this case with the Learner, is likely to reduce the number of shocks, helping to explain the results for the Woman condition.

In Milgram’s experiment 13^3^ it was arranged that the experimenter is an ‘ordinary man’ rather than a scientist. In this case the legitimacy of the experimenter was challenged, through the experimenter apparently being another subject (in fact a confederate). The degree of obedience in this condition dropped dramatically (to 4/20 complying). In the current experiment it can be argued that the legitimacy of the virtual experimenters could also be challenged. The week before a group like them (though not wearing the white coats) had been sitting around a bar harassing a woman. Those in the Woman condition experienced this harassment aimed at themselves. What legitimacy did these men have to run a memory learning experiment, moreover using the same types of comments as in the bar talk (that women are always complaining etc)? There is some evidence for this in the frequency analysis of the descriptions by the participants about their experience after Phase 2 of the Bar scenario. Some, only in the Woman condition, reported that the men were ‘idiots’.

In their analysis of the Milgram audio tapes Rochat and Modigliani^67^ point out that an important distinction between the two conditions was that in the Learner as Stranger condition there was a possible identity between the subject and experimenter, signified most especially by subjects tending to mimic the behaviour of the experimenter – for example, by interrupting and talking over the Learner. However, in the Friend condition the opposite was the case – there was a strong identification with the Learner, with the subjects talking directly to him, rather than ignoring and talking over him. Imitation of the experimenter in the Stranger condition was associated with fully obeying him. Whether this was identification or compliance, or both, is unresolved, although people can be compliant without being imitative, and it is known that imitation can signal rapport^68^. However, the crucial point is that identification with the victim appeared to be a critical element in allowing subjects to resist the demands of the experimenter, and disobey. We put forward the view that in our experiment, the identification with the virtual Learner came from having been embodied in a similar situation the week before, being verbally attacked by a group of hostile men (with the same facial appearance and voices as the virtual experimenters). In the Group condition, however, participants received a double dose of being in-group with the men. The Group condition was ideal to set up an in-group identification with the men in the Shock phase (‘Women are always complaining’) whereas the Woman condition was more likely to establish an in-group identification with the woman. The identification here is not that ‘I am a friend of that person’ but ‘I was that person’. Reicher, *et al*.^7^ support the identification explanation of obedience – subjects will follow through their identification with science represented by the experimenter. This theme was also taken up in^12^.

In Milgram’s experiment 13 A^3^, when it was arranged that the experimenter himself became the Learner, no subjects at all agreed to continue shocks once the Learner had said that he wanted to stop. Indeed some subjects physically restrained the other confederate who was insistent on continuing the shocks. This also supports the identification explanation. While the experimenter acting as experimenter insists that the experiment continue (invoking the cause of science) subjects tend to obey, but when the experimenter (as victim) declares an end to the shocks, subjects go out of their way to ensure that the experimenter’s wish is fulfilled. Note that in this condition, with experimenter as victim, all subjects stopped as soon as the victim demanded it (at 150 v). This is similar to our result, where in the Woman condition that moment when the victim started to vociferously complain was the most important bifurcation point, when participants either stopped (the majority in the Woman condition) or continued.

The evidence suggests that those in the Group condition identified with the experimenters, with whom they had been in the same in-group a few days before. This is how they could administer the shocks with a decreased HR compared to the other conditions. Our data suggests that this decreased HR was associated with a lessening of subjective physiological arousal, which in turn was associated with less Plausibility. Further research is needed to test the model that emerged from these data, as expressed in Eq. 4: the key is physiological arousal, greater arousal leading to fewer shocks, or lesser arousal leading to more shocks, but mediated through Plausibility. Essentially, from the point of view of the participant it would seem as though: ‘In this situation I should be feeling aroused as I am giving these shocks to someone who wants to me stop. Since I do not feel so aroused, this can’t really be happening, therefore I will continue to give the shocks.’

There are several limitations of the study that require further work. First, the Learner was depicted as a woman. It would be interesting to know whether the same results would have been found had the virtual Learner been shown as a man. If not then this would lend weight to the supposition that the results were specifically due to the harassment of the woman. If the results were the same then it would support the idea that just the fact of having been a victim would be enough to influence the outcome in the Shocks scenario. Second, the finding that Plausibility influences whether or not participants fall into the high or low shocks group was unexpected prior to the study. A further study would be needed to explicitly test the possible causal chain that we have suggested from HR change through subjective assessment of physiological response affecting Plausibility in turn affecting the number of shocks. Third, we do not know how results would be influenced had the participants thought that the Learner was a real-time representation of an actual person who was feeling the shocks. This is ethically problematic, since it would involve deception. In a VR simulation^69^ of the Asch experiment mentioned in the introduction there is some evidence supporting the finding that participants did show conforming behaviour whether the peer group were avatars or believed to be representations of real people. However, using robots rather than characters in VR, in another reconstruction of the Asch conformity experiment, it was found that the robots did not influence the judgements of subjects^70^. This remains an open question. Fourth, while embodied as the Woman the movements of the character, except head movements, were prescripted and the utterances were those of the woman. It would have been interesting to know what would have happened had the participants had full agency, able to say whatever they wanted back to the harassers rather than witnessing a replay of the virtual woman’s statements. This would require voice transformation from male to female, but such technology is increasingly available. Fifth, our evidence that embodiment as the woman was effective in reducing the number of shocks is interesting, but not enough. We do not know whether this would influence their subsequent behaviour in real life, especially in regard to their interaction with women. It would be possible, to obtain long term information on how subsequent behaviour had changed, or even recall participants, for example, one year later, and expose them to a VR scenario that involved sexual harassment. Sixth, we had not foreseen the impact of two exposures to the Group condition influencing the number of shocks in the way that this turned out. With hindsight all participants in the Group and Control conditions should have experienced the Woman condition at the end of collecting all their data.

Following on from this, a cautionary aspect to these results is that they are double-edged. Although the number of shocks administered in the Woman condition decreased compared to the Control condition, the number of shocks given by those in the Group condition clearly increased compared to the controls. As reported above we took further steps in response to this finding. It should therefore be noted that the same technology that can be used for beneficial purposes can also be used for negative purposes, with equal effect. The ethical issues surrounding the general area of virtual embodiment have been considered in depth by Madary and Metzinger^71^, and recently by Slater, *et al*.^72^ and as this technology and its application develop these issues will become more and more urgent. We have learned through this study that it is vitally important to be aware of possible negative outcomes to experiences in this field, and take precautions against those, even if such outcomes are unlikely *a priori*. Although we think of VR being used for beneficent personal and social outcomes – indeed there are a vast number of studies in the past 30 years where VR has been used in the context of psychological therapy^73,74^ – we also have to be cautious that sometimes in the pursuit of a positive outcome, a corollary might be a negative outcome, notwithstanding the wishes of the scientists involved.

This research was initiated by a question posed by the U.S. Office of Naval Research: there are circumstances when a group of military personnel might engage in illegal and immoral acts against others, and each person participate because of the overall group solidarity and individual conformity. Are there ways to combat this? The answer appears to be in the affirmative. By allowing group members to take part in such a virtual scenario, and then live through the consequences of their own actions as a victim, they may become aware of the illegitimacy and immorality of the behaviour of the perpetrators, and thus avoid engaging in such behaviour. Of course, this is a hypothesis which although based on our results needs to be addressed by further experimental work.

## Supplementary information

Supplementary Info.

Data S1.
