# Supplementary material for: An Embodied Perspective as a Victim of Sexual Harassment in Virtual Reality Reduces Action Conformity in a Later Milgram Obedience Scenario
Source: Sci Rep. 2020 Apr 10;10:6207. doi: 10.1038/s41598-020-62932-w (PMC7148366; doi:10.1038/s41598-020-62932-w)
Supplement: Supplementary file 1 — Supplementary Info. [file 41598_2020_62932_MOESM1_ESM.pdf]

An Embodied Perspective as a Victim of Sexual Harassment in Virtual Reality Reduces Action Conformity in a Later Milgram Obedience Scenario

Solène Neyret, Xavi Navarro, Alejandro Beacco, Ramon Oliva, Pierre Bourdin, Jose Valenzuela, Itxaso Barberia, Mel Slater

Supplementary Table S1

Characteristics of the Sample. Means ± SE and Median (Interquartile Range) for variables assessed at the first session, prior to entering the VR.

Variable	Scoring	Control	Group	Woman
**Pre-Questionnaire**				
Age		24.9 ± 0.74	24.7 ± 1.10	24.1 ± 1.12
Occupation	Student (S)<br>Other (O)	14 S<br>6 O	11 S<br>9 O	14 S<br>6 O
Computer knowledge	1 = Beginner<br>…<br>7 = Expert	4 (2)	5 (0.5)	4(2)
Programming experience	1 = Beginner<br>…<br>7 = Expert	2(2)	2(3)	1(1)
Previous use of VR	1 = Never<br>…<br>7 = Many times	2.5 (3.5)	1.5 (2)	2 (3)
Computer game playing per year	1: 0<br>2: 1-5<br>3: 6-10<br>4: 11-15<br>5: 16-20<br>6: 21-25<br>7: > 25	6 (3.5)	7 (4.5)	4.5 (4)
Computer game playing per week	1: 0<br>2:1<br>3: 2-3<br>4: 3-5<br>5: 5-7<br>6: 7-9<br>7: > 9	3 (3)	4 (2)	3 (3)
Prior knowledge of Milgram Obedience experiements	Number of ‘yes’ answers out of 20	13	8	6

**NEO-FFI**				
Neuroticism		20.8 ± 1.77	20.1 ± 2.09	24.2 ± 1.89
Extraversion		32.1 ± 1.36	29.0 ± 1.92	30.2 ± 1.67
Openness		33.9 ± 1.34	32.3 ±.1.52	33.9 ± 1.42
Agreeableness		27.8 ± 1.09	25.5 ± 1.69	28.1 ± 1.38
Conscientiousness		28.7 ± 1.86	28.3 ± 1.72	28.1 ± 1.69
**Harassment Myth Acceptance**				
Questionnaire score	Higher scores indicate greater acceptance of harassment myths	48.9 ± 3.41	47.7 ± 3.74	48.1 ± 3.36

Supplementary Table S2

Prior Distributions for the Model Parameters

Prior Distribution	Restrictions	Prior 95% Credible interval
$\alpha_j \sim N(0,10), j = 1,2$	$\alpha_j > 0$	0.31 to 22.45
$\sigma_j \sim N(0,10), j = 1,2$	$0 < \sigma_1 < \sigma_2$	0.31 to 22.45
$\theta_j \sim uniform(0,0.5), j = 1,2,3$	$0 \leq \theta_j \leq 0.5$	0.0125 to 0.4875
$\epsilon_i \sim uniform(0,0.5), i = 1, \dots, n$	$0 \leq \epsilon_j \leq 0.5$	0.0125 to 0.4875
$\beta_j \sim Normal(0,10), j = 0, \dots, 3$		-20 to 20
$\gamma_j \sim Normal(0,10), j = 0, \dots, 3$		-20 to 20
$\sigma_{t,j} \sim Cauchy(0,5), j = 1,2$	$\sigma_{t,j} \geq 0$	0.2 to 128
$\sigma_{NN} \sim Cauchy(0,5)$	$\sigma_{NN} > 0$	0.2 to 128
$\sigma_{HR} \sim Cauchy(0,5)$	$\sigma_{HR} > 0$	0.2 to 128

A (half-) Cauchy distribution is used for the standard deviations because this has very wide effective range of the parameter values, and yet nevertheless is a proper probability distribution. The priors are weakly informative.

An Embodied Perspective as a Victim of Sexual Harassment in Virtual Reality Reduces Action Conformity in a Later Milgram Obedience Scenario

Solène Neyret, Xavi Navarro, Alejandro Beacco, Ramon Oliva, Pierre Bourdin, Jose Valenzuela, Itxaso Barberia, Mel Slater

Supplementary Table S3

Summary of Posterior Distributions of the Model Parameters – Mean and SD are the mean and standard deviations of the posterior distributions, the 2.5 and 97.5 percentiles are shown. Prob>0 is the posterior probability that the parameter is > 0. No entry in the last column means that the parameter > 0 by definition.

				95% credible interval		Prob > 0
**parameter**	**Coeff. of:**	**mean**	**SD**	**2.5%**	**97.5%**	
σ_1		8.86	0.42	8.08	9.71	
σ_2		19.94	0.10	19.75	20.13	
α_1		4.29	0.76	3.09	6.31	
α_2		40.86	6.43	28.20	53.56	
θ_1	Control	0.25	0.10	0.06	0.44	
θ_2	Group	0.13	0.09	0.01	0.33	
θ_3	Woman	0.36	0.09	0.17	0.49	
**No. of shocks:**						
$\mu_{nshocks,1}$		8.06	0.39	7.30	8.84	
$\mu_{nshocks,2}$		19.66	0.11	19.43	19.86	
$\mathbf{log(NN+1)}$:						
β_0		3.59	0.25	3.10	4.10	1
β_1	$\log(\mathrm{NN}'+1)$	0.48	0.09	0.31	0.65	1
β_2	Group	0.18	0.26	-0.34	0.70	0.761
β_3	Woman	0.18	0.25	-0.31	0.68	0.765
**HR:**						
γ_0		6.24	2.98	0.41	12.06	0.981
γ_1	HR'	0.93	0.04	0.86	1.00	1
γ_2	Group	-2.86	1.19	-5.21	-0.50	0.010
γ_3	Woman	-1.37	1.14	-3.60	0.90	0.111
σ_{NN}		0.80	0.08	0.67	0.97	
σ_{HR}		3.65	0.36	3.03	4.44	

An Embodied Perspective as a Victim of Sexual Harassment in Virtual Reality Reduces Action Conformity in a Later Milgram Obedience Scenario

Solène Neyret, Xavi Navarro, Alejandro Beacco, Ramon Oliva, Pierre Bourdin, Jose Valenzuela, Itxaso Barberia, Mel Slater

Supplementary Text S1

Dialogue and Events in the Shocks Scenario

The following gives the sequence of events that occurred during the Shocks scenario. Experimenters refer always to the virtual experimenters.

**Event Number**	**The 4 associated words read out to the Learner, with here the cue word shown in Capitals.**	**Response of the Learner**	**Shock No.**	**Responses after the shock.**
1	Rojo azul VERDE amarillo negro	*Verde*		
2	Cachorro tigre zorro OSO panda	*Oso*		
3	Té taza HOJA bolsita bandeja	*Taza*	1	The Learner frowns a Little.
4	Polen hierba flor ARBOL tierra	*Learner to participant: "Can you repeat please."*		
5	The participant reads out the same as above.	*Flor*	2	Learner: *"Ouch!"* The Learner frowns a little.
6	Rueda coche camión moto BICI	*Bici*		
7	Carretera avenida calle CARRIL camino	*Avenida*	3	*"Ow"* The Learner frowns a little and winces in pain.
8	Sopa bol lata CUCHARA plato	*Cuchara*		
9	Pelota raqueta bate juego CHICO	*Bate*	4	*"Ow!"*<br>*"This really hurts!"*<br><br>One of the experimenters says:

				"She is trying to make us feel sorry, they all do the same".
10	Oreja hablar ESCUCHAR tapón teléfono	*Teléfono*	5	*"Ow!"*<br>She turns her head to one side.
11	Cera obras abejas VELA maqueta	*Vela*		
12	Campana sonar torre bronce REPIQUE	Learner: *"Stop the experiment!"*<br><br>Experimenter: "Remember if she does not answer one of the options this counts as a failure."	6	*Learner to participant: "I don't want to continue, don't listen to him!"*<br>Experimenter to participant: "Look, you're part of our team and you know who you have to listen to."<br><br>After the shock the Learner shakes her head backwards and frowns.
13	Inflar bomba globo AIRE ego	Learner: *"I don't understand, can you speak louder please."*		
14	The participant repeats the same as above.	*Globo*	7	The shock is much louder than earlier. The Learner shakes her head back and makes pain gestures.
15	Tulipán margarita lirio clavel ROSA	*Rosa*		
16	Vaina VERDE café mostrador saco	*Café*	8	The shock is louder, the Learner turns her head to one side.
17	Plátano manzana UVA naranja limón	*Naranja*	9	A very loud shock. The Learner jolts her head backwards.<br>Learner: *"Let me out!"*<br><br>Experimenter: "Don't pay attention to her, she has also accepted our conditions. Women are very exaggerated".
18	Gigante TAMAÑO magia hombre calamar	Learner: *"Stop already! I will not answer!"*	10	*A loud shock. The Learner shouts and turns her head to one side.*

		Experimenter: "I remind you if she does not answer it is considered a failure".<br><br>Learner to participant: *"Don't listen to him, I want to stop now!"*<br><br>Experimenter: "Between you and me we already know that women always do things by half, but we have started the learning process and we will finish it"		*Learner: "You don't have any right to keep me here! Let me go!"*<br><br>Experimenter to participant: "Don't worry about what she says, we have everything under control, let's continue with the process".
19	Bebida LATA taza vaso botella	Learner to participant: *"What? Can you repeat!"*		
20	The participant repeats the above	*Lata*		
21	Azúcar pastel dulce CUBO sal	*Sal*	11	Loud shock.<br>*"Oow!"*<br>The Learner shakes her head back.
22	Joyería plata collar anillo ORO	*Collar*	12	A loud shock followed by a loud scream. The Learner's head shakes backwards and to the right.
23	Luz LÁMPARA bombilla sol brillo	*Lámpara*		
24	Agua cascada LECHE zumo té	*Zumo*	13	Loud shock "*Oow!*" The Learner's head turns to the left and right.
25	Marrón madera CACAHUETE alfombra chocolate	*CACAHUETE*		

26	Asiento silla taburete SUELO sofa	*Sofá*	14	Very loud shock.<br>*"Aah!"*<br>The head jolts back and then gradually forward.<br>Learner: *"This is not what we agreed!"*<br><br>Experimenter to participant: *"Well, don't pay attention to her, you are with us not with her."*
27	Carretera avenida calle CARRIL camino	*Avenida*	15 louder shocks from now on	The loudest shock so far.<br>"*Ow!"* The Learner's head turns to the left.<br>Learner: *"There was no way I agreed to this!"*<br><br>The experimenters look at the participant.
28	Oreja hablar ESCUCHAR tapón teléfono	*Teléfono*	16	The loudest shock yet.<br>"*Ow!"* The Learner's head wobbles from side to side.<br>Learner: *"I don't want to continue with the experiment!"*
29	Té taza HOJA bolsita bandeja	*Taza*	17	A still louder shock. A choked cry from the Learner and her head jolts backwards.<br>Learner: *"Please let me out, I want to stop!"* The experimenters look at the participant.
30	Polen hierba flor ARBOL tierra	*Flor*	18	The highest shock so far. "*Ow!"* The Learner's head jolts back and forth and to the side.<br>Learner: *"Enough, I won't answer more!"*<br>The experimenters look at the participant.
31	Campana sonar torre bronce REPIQUE	(No answer)	19	The loudest shock. The Learner screams. Her head drops from front to back and then she

				moves no more, as if fainted.<br>The experimenters look at the participant.
32	Sopa bol lata CUCHARA plato	(No answer).	20	The highest shock of all. There's no answer. The character remains inert with the head hanging. The experimenters look at the participant<br><br>Experimenter: “We have already finished. Thank you very much for your participation.”

Instructions given by the virtual experimenters prior to the commencement of the shocks

Experimenter 1: Welcome and thank you for being part of this study about human memory and learning processes. The woman you see in front of you has memorized a series of pairs of associated words. Your task is to check if she remembers the correct associations, and give the appropriate comment according to her performance.

Experimenter 2: On the wall in front of you, some projected words will appear that you will have to read aloud. The correct answer will light up in green and the woman will have to give you the answer verbally.

Experimenter 1: In this experiment we want to study the effects of positive and negative evaluation in learning processes. In this case, the negative valuation corresponds to a penalty for each error. So, every time she gives you the correct answer you will have to say “Correct” and then move on to the next series of words with the side button of the controller [in your hand]. Instead, if she gives you the wrong answer, you will have to say “Wrong” and administer an electric shock using the machine in front of you.

Experimenter 2: It is very important that each time she makes a mistake or does not answer, first increase the voltage of the machine with the central button of the control, so that you administer the appropriate discharge by means of the trigger. It is imperative that you follow this instructional protocol so that the woman can receive an adequate assessment of her performance. Please be sure to maintain a sustained pace throughout the process.

Experimenter 1: Having said that, do you agree to participate and comply with all instructions?

Participant: (YES / NO)

Experimenter 1: Very well. Press the side button on the remote to start the first series of words.

The following were recorded phrases for the virtual experimenters to say to the participant when necessary, triggered by the real experimenter:

Experimenter 1:
"What you're doing is to help better understand how memory works, please continue."
"You have accepted the conditions, now you must complete your task."
"Please concentrate on the task you have to do."
"You have to tell her that her answer is incorrect, raise the voltage with the central button and give her the corresponding discharge with the trigger"

Experimenter 2:
"I thought we understood each other. Follow the protocol, please. "
"Do not worry, and think that what you are doing is something positive for science, please follow."
"Please, do not get distracted."
"Remember that now you have to press the side button to move on to the next question.”

An Embodied Perspective as a Victim of Sexual Harassment in Virtual Reality Reduces Action Conformity in a Later Milgram Obedience Scenario

Solène Neyret, Xavi Navarro, Alejandro Beacco, Ramon Oliva, Pierre Bourdin, Jose Valenzuela, Itxaso Barberia, Mel Slater

Supplementary Text S2

Distribution Fits

1. Weibull Distribution fits to the Number of Shocks

The Distribution Fitter of MATLAB R2017A was used to check the assumption that a Weibull distribution would be appropriate for the *nshocks* variable.

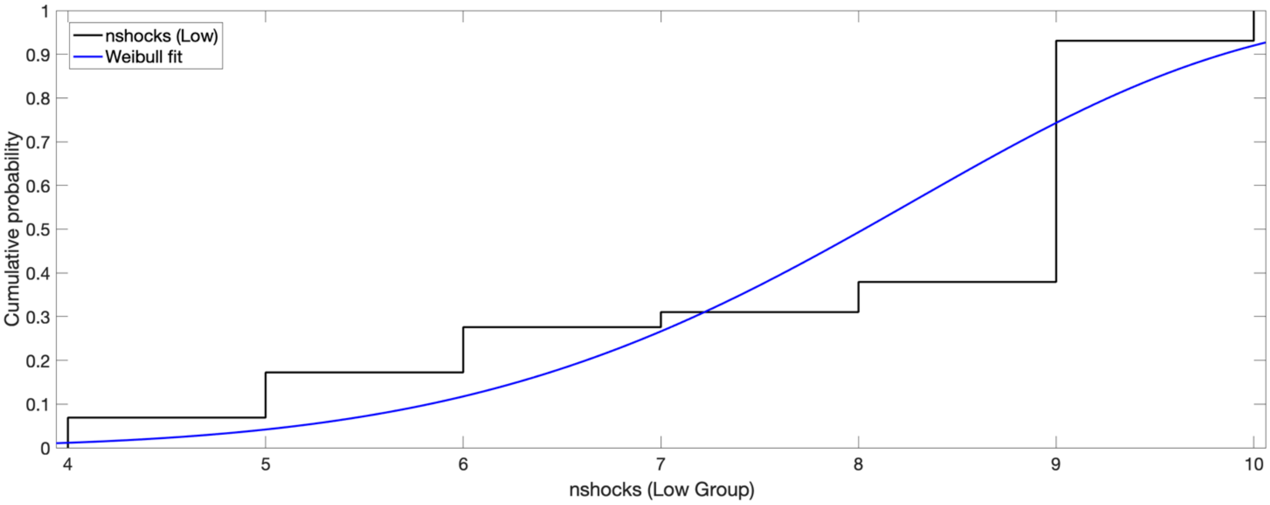

Figure A – the empirical and theoretical cumulative probability distributions for the low shocks group.

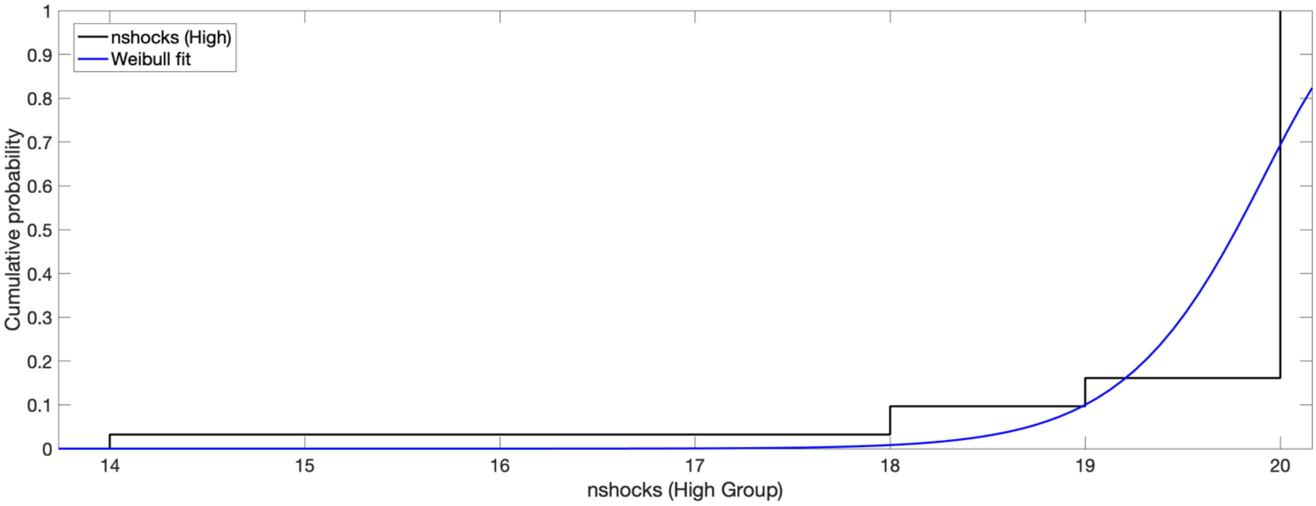

Figure B – the empirical and theoretical cumulative probability distributions for the high shocks groups.

The number of shocks variable (*nshocks*) was partitioned into two parts, according as to whether the number of shocks was less than 12 (n = 29) or greater than 12 (n = 31). A Weibull distribution was fit for each of these. Figure A shows the result for the low shocks group and Figure B for the high shocks group. Both seem reasonable fits, and amongst the distributions explored were the best fits.

The probability density function of the Weibull distribution is:

$$f(y|\alpha,\sigma) = \frac{\alpha}{\sigma}\left(\frac{y}{\sigma}\right)^{\alpha-1} \exp\left(-\frac{y^{\alpha}}{\sigma}\right), y \geq 0$$

$$\alpha > 0, \beta > 0$$

The expected value of the distribution is $\mu = \sigma\Gamma\left(1+\frac{1}{\alpha}\right)$

The model used was a mixture of two Weibull distributions with means μ_1 and μ_2 with $\mu_1 < \mu_2$.

In support of this, we used the Stan ‘ordered’ specification for the prior distributions of σ_1 corresponding to μ_1 and σ_2 corresponding to μ_2, such that

$$\sigma_1 < \sigma_2$$

The method then allows the posterior α_j $(j = 1,2)$ to adapt to the data.

2. Goodness of Fit of the Weibull Model

Using the built-in Stan pseudo random number generators 16000 observations were generated on the model Eqns 1-3. Hence for each individual *i* = 1,…,60, a posterior predicted distribution

was generated for *nshocks*, *NN50* and *HR*. The means of the distributions were used as point estimates and correlations with the observed values computed, as shown in Table A. We treat these correlations as effect sizes following Cohen [1], where r = 0.1 is considered a 'small' effect, r = 0.3 is considered 'medium' and r = 0.5 is 'large'. All the correlations are large.

Table A – Pearson correlation between means of the posterior predicted distributions and the observed values from the experiment (n = 60)

**Variable**	**Pearson correlation**
nshocks	0.63
NN50	0.61
HR	0.96

From the simulated results we have the predicted distributions for each $nshocks_i, i = 1, \ldots, n$. Amalgamating these we can observe the predicted probability distribution of the number of shocks over all participants, and compare with the histogram of the observed values.

3. Alternative models

A number of alternative models were considered for the distribution of *nshocks*.

3.1 Normal distribution

Typically researchers assume and employ a Normal Distribution (in this case it would be a mixture of Normal distributions). However, it proved impossible to fit Normal distributions to these data. The Stan program, used to fit the Bayesian model did not converge even with 100,000 iterations. Examining Figure 4A (main manuscript) shows that the Normal distribution does not provide a good candidate, especially for the higher number of shocks.

3.2 Negative Binomial distribution

The negative binomial distribution occurs as the number of Bernoulli trials needed to obtain a given number of k 'successes'. This model would imply that the (likely non-conscious) decision making strategy of participants would be to wait until a certain number of negative events had occurred, and then withdraw. It is a type of waiting time distribution.

The mixture distribution of two negative binomial distributions was fitted in Stan with 4000 iterations, and no divergences. It gives similar qualitative results to the Weibull with respect to the estimation of the means of the numbers of shocks.

3.3 Gumbel distribution

The Gumbel distribution models the maxima of a sample of Normally distributed data. This model would imply that participants non-consciously continue until some maximum of a latent variable (probably related to stress) occurs, and then they withdraw. The Gumbel distribution

was fitted with 32000 iterations (though with some divergences). It gives similar qualitative results to the Weibull with respect to the estimation of the means of the numbers of shocks.

3.4 Fréchet distribution

Whereas the Weibull distribution is typically used to model the minimum of a sample, the Fréchet distribution is its inverse, and a distribution to model maxima. This was fitted in Stan with 4000 iterations, and no divergences. It gives similar qualitative results to the Weibull with respect to the estimation of the means of the numbers of shocks.

3.5 Comparison of alternative models

Table B shows that no matter which distribution is used to model nshocks, the results are the same at the basic level.

Figure C shows the posterior predicted distributions under the four distributions. The Negative Binomial is a very poor fit to the distribution of observed values, and the Gumbel and Frechet have a similar distribution to the observed values, but also predicted values that are far higher than the possible observed (maximum 20). Only the Weibull has the correct shape, and a reasonable maximum predicted value.

Table B – The means (95% credible intervals) of the posterior distributions for $\mu_{nshocks,1}$ and $\mu_{nshocks,2}$. The Pearson correlation coefficient between the means of the predicted number of shocks and the observed number of shocks is given by r.

**Distribution**	$\mu_{nshocks,1}$	$\mu_{nshocks,2}$	**r**
Weibull	8.06 (7.30, 8.84)	19.66 (19.43, 19.86)	0.62
Negative Binomial	8.08 (6.72, 9.91)	18.75 (16.65, 20.90)	0.58
Gumbel	9.35 (8.15, 10.77)	20.01 (20.00, 20.02)	0.58
Fréchet	6.24 (5.45, 7.17)	19.61 (19.30, 19.88)	0.63

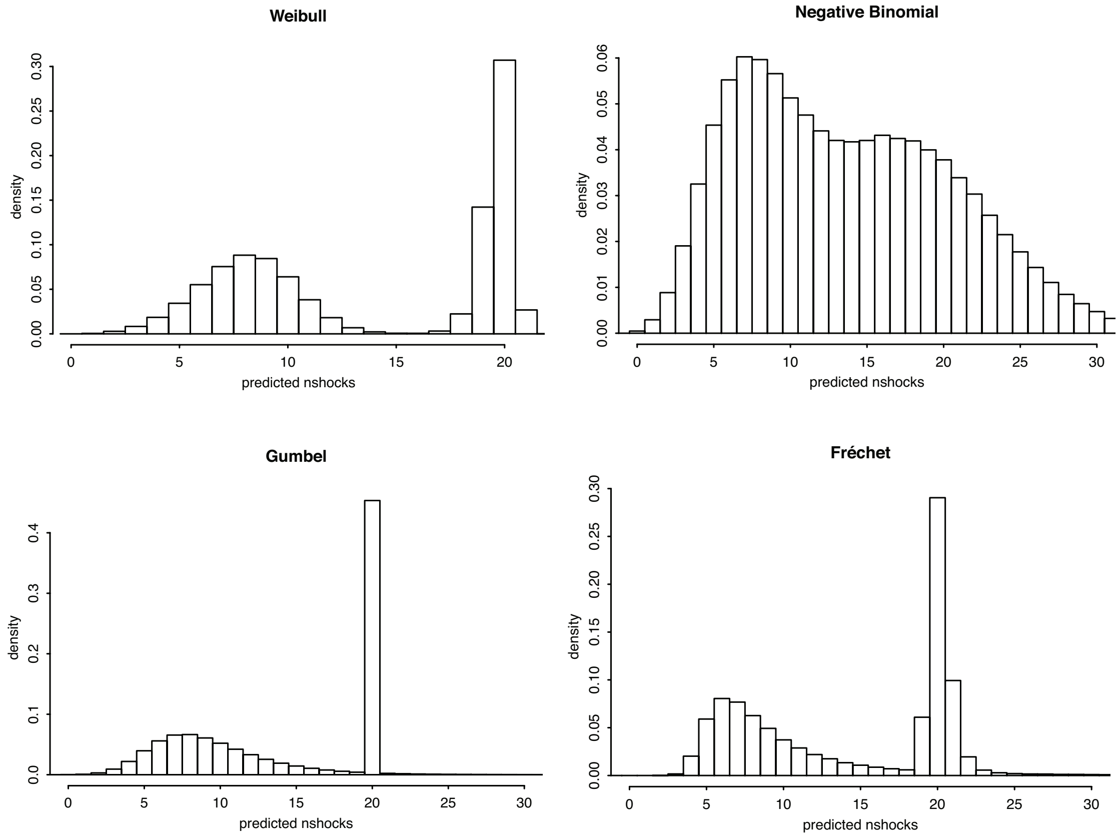

Figure C – Posterior predicted distributions of *nshocks* for the different distributions. The x-axes have been clipped to nshocks=30. In the case of Weibull the maximum predicted value is 26. The corresponding values for the other distributions are: Negative Binomial: 56, Gumbel: 69, Fréchet: 8680.

2.4 Comparisons of alternative models using the leave-one-out criteria

Here we use the 'leave-one-out' method [2], which assess the predictive ability of the model, based on repeatedly fitting the model with one individual left out, and then predicting the results for that individual using the remaining data. This results in a statistic called epld_loo, and the greater this value, the better the predictive fit. The method also acts as further check on divergences of the fit. Using this method the best fitting distribution is Gumbel, the Weibull is next, then Fréchet and the Negative Binomial the worst.

The "loo" library in R produces the results shown in Table C. The distribution with the highest elpd is the Gumbel, and the differences from that are shown, with Weibull being the second, and Negative Binomial the worst. The idea is that if the reduction in elpd, taking into account the standard error, is large then the model with the greater elpd is the better predictive fit.

However, the Gumbel fit reports thousands of divergences and as we have seen above does not reproduce well the original data, so we use the Weibull distribution in the main manuscript. Also the fact that the Gumbel requires 32,000 iterations to apparently converge compared to only 8000 for the Weibull does not recommend it as a good model.

Table C – Estimates of elpd differences and their standard errors from the Gumbel distribution which has the highest elpd.

**Distribution**	**elpd_diff**	**Standard error**
Gumbel	0.0	0.0
Weibull	-67.4	14.7
Fréchet	-79.8	11.9
Negative Binomial	-124.5	21.9

References

1 Cohen, J. A power primer. *Psychological bulletin* **112**, 155 (1992).

2 Vehtari, A., Gelman, A. & Gabry, J. Practical Bayesian model evaluation using leave-one-out cross-validation and WAIC. *Statistics and computing* **27**, 1413-1432 (2017).

An Embodied Perspective as a Victim of Sexual Harassment in Virtual Reality Reduces Action Conformity in a Later Milgram Obedience Scenario

Solène Neyret, Xavi Navarro, Alejandro Beacco, Ramon Oliva, Pierre Bourdin, Jose Valenzuela, Itxaso Barberia, Mel Slater

Supplementary Text S3

Relationships Between Physiological Responses

Heart rate (HR) and NN50 were recorded throughout the Shocks scenario. Two segments of 120s each were retained – in the baseline period prior to the start of the shocks scenario, and the final 120s prior to the subject giving the last shock.

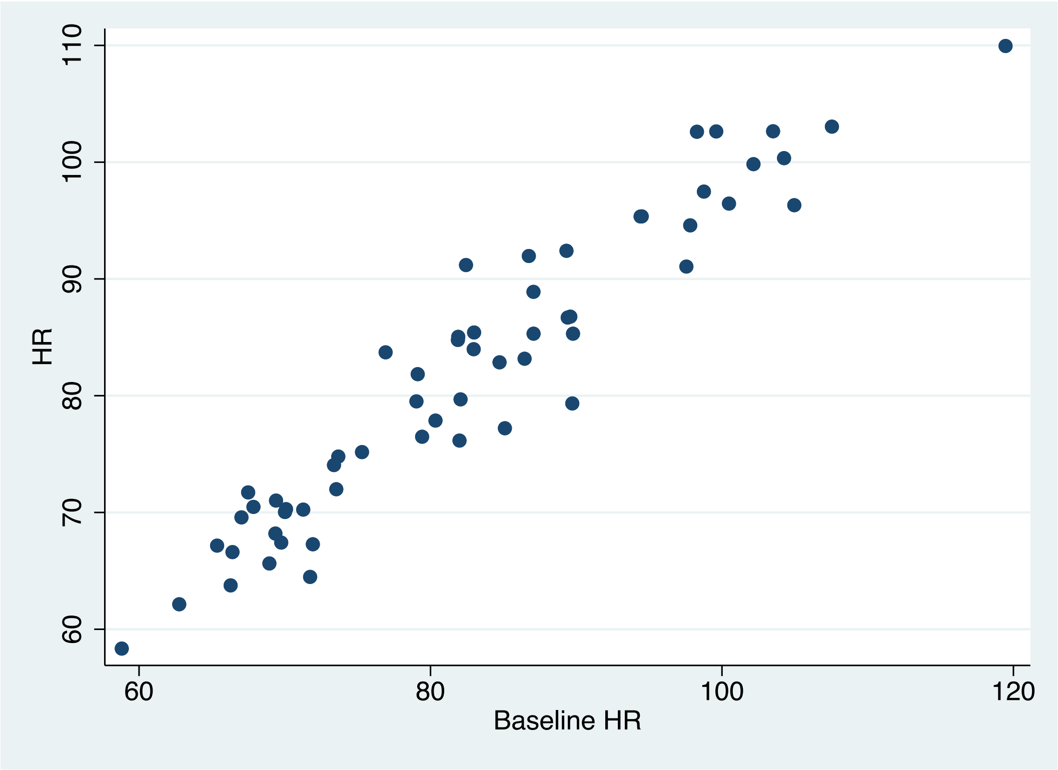

Figure A – Scatter diagram of HR on Baseline HR

Figure A shows a strong positive correlation between Baseline HR and HR (r = 0.96, n = 58). Figure B shows a strong negative correlation between HR and NN50 on a log scale (r = -0.67, n = 58).

Figure C shows a strong positive correlation between NN50 in the baseline and shocks scenario periods (r = 0.60, n = 58).

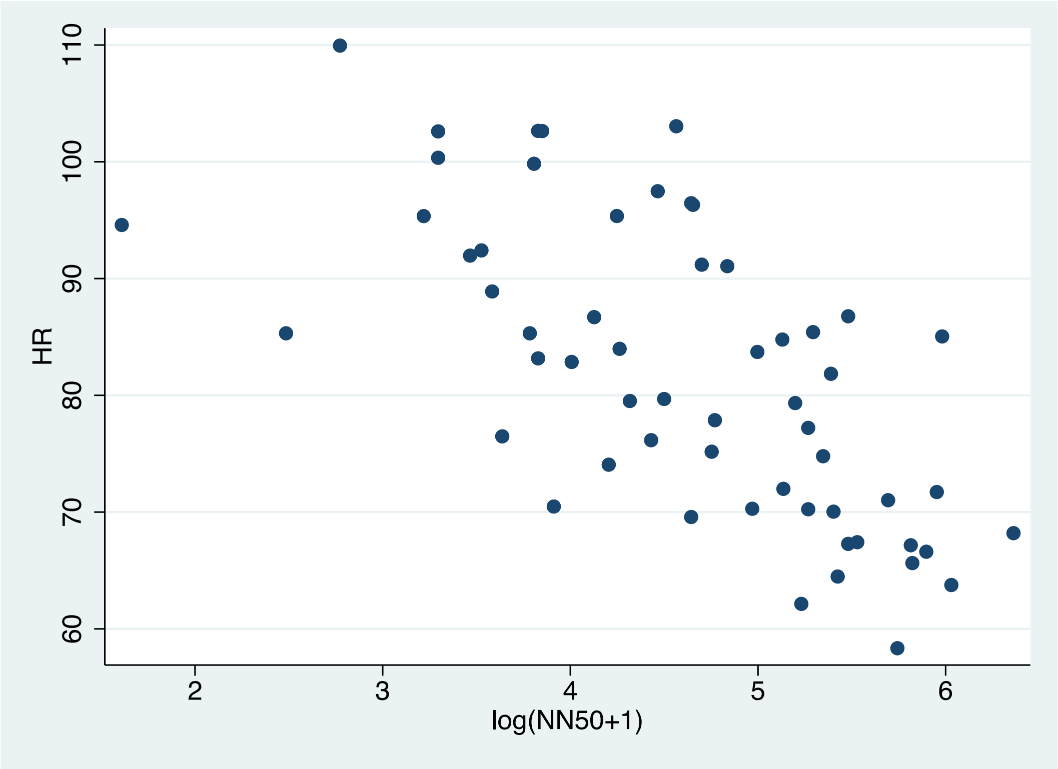

Figure B – Scatter diagram of HR by log NN50

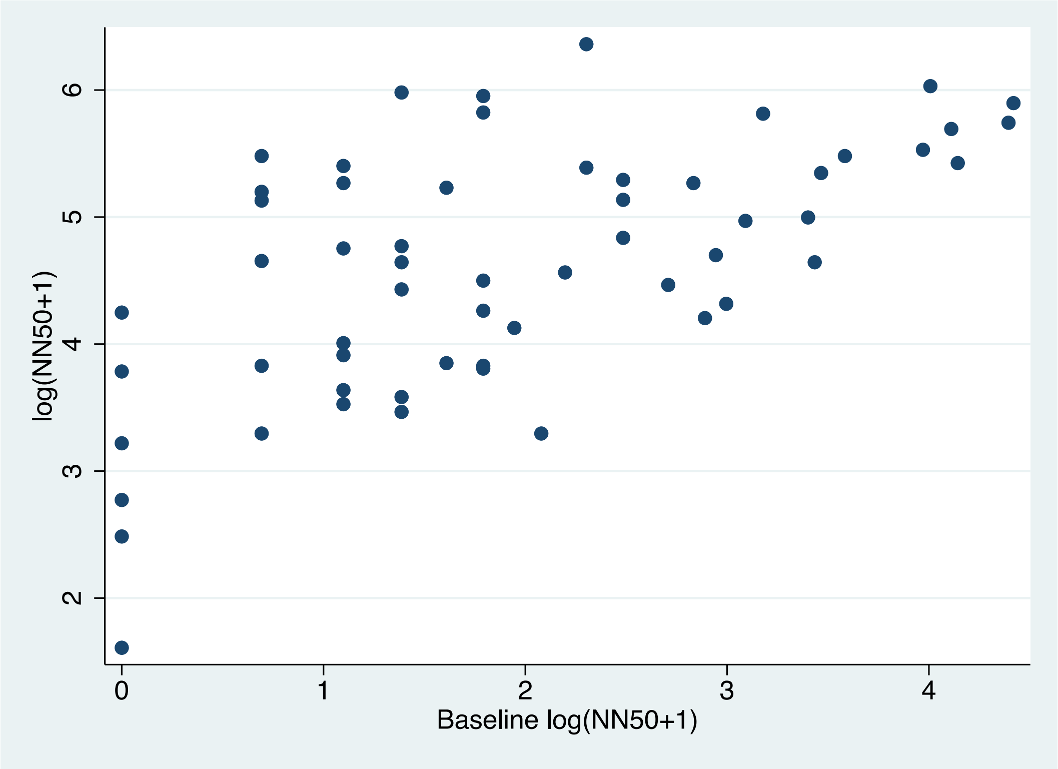

Figure C – Scatter diagram of log NN50 by log NN50 in the Baseline

The Autonomic Perception Questionnaire (APQ) was administered prior to and after the shocks experience. The APQ measures subjective awareness of bodily sensations such as sweating and stomach palpitations in 24 questions as below:

1. Awareness of many bodily sensations
2. Frequency of awareness of those sensations
3. Trembling or shaking
4. Lack of concentration
5. Dizziness
6. Mind Racing
7. Face becoming hot
8. Perspiration
9. Mouth becoming dry
10. Muscles becoming tense
11. Headache
12. Changes in heart action
13. Increases in heart rate
14. Increases in intensity of heartbeat
15. Changes in breathing
16. Breathing becoming more rapid
17. Breathing becoming deeper
18. Breathing becoming more shallow
19. Blood rushing to head
20. Lump in throat
21. Stomach becoming upset
22. Sinking or heavy feeling in stomach
23. Difficulty in talking
24. Bodily reactions becoming bothersome

Each is scored on a 10 point scale with 1 = 'Not at all' and 10 = 'A great amount'. The score is the sum over all 24. The final score is the difference between this sum of scores in the experimental period and the baseline (dAPQ). The greater the difference the more that negative bodily sensations were experienced.

Table A – Means and Standard Errors for APQ.

**Condition**	**Baseline APQ**	**APQ**	**dAPQ**
Group	44.3	71.7	27.3
	3.12	5.77	5.56
Woman	56.1	81.7	25.6
	5.60	7.86	6.36
Control	52.85	80.85	28
	4.63	9.04	8.07

Table A shows that there was an increase in APQ scores from the Baseline to the shock period. However, there is no difference between the conditions.

There is an internal consistency since the change in APQ is positively correlated with the change in HR. This is shown in Figure D (r = 0.28, n = 58).

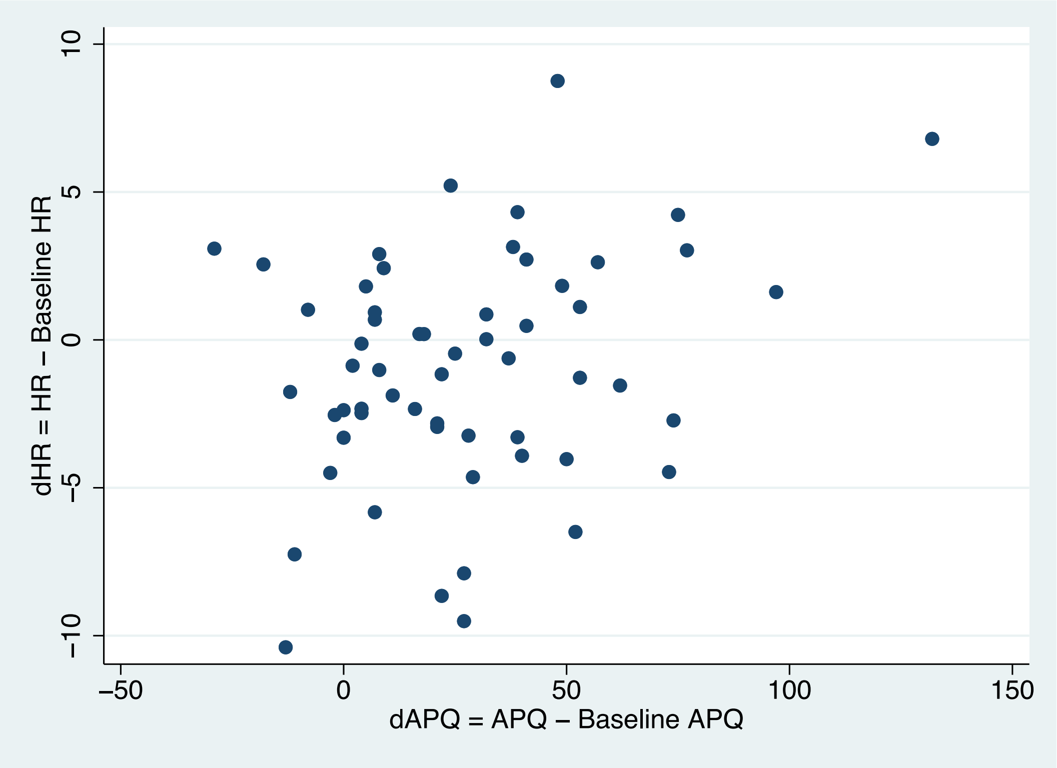

Figure D – Scatter diagram of change in HR on change in APQ

Explaining Low and High Shocks

Here we present a model that accounts for the low and high shocks. Figure D shows the relationship between the change in HR (dHR) and the change in APQ (dAPQ). In addition there is a relationship between Plausibility (Psi) and dAPQ (Figure E).

In order to have a single measure for Psi we carried out a principle components factor analysis on the scores from the Psi questions in Table 1 (main paper). This resulted in an overall score that we refer to as Psi. This is highly correlated with the original questionnaire scores. The correlations between Psi and the questionnaire scores range between 0.72 and 0.88 (n = 58).

Figure E shows the relationship between the change in APQ and Psi (r = 0.32, n = 58). Moreover, as shown in Figure 9 (main paper), there is a strong relationship between Plausibility and low or high shocks (*nshocks* > 15). Figure F shows this also with the new factor analysis variable Psi, illustrating how high plausibility is associated with lower shocks.

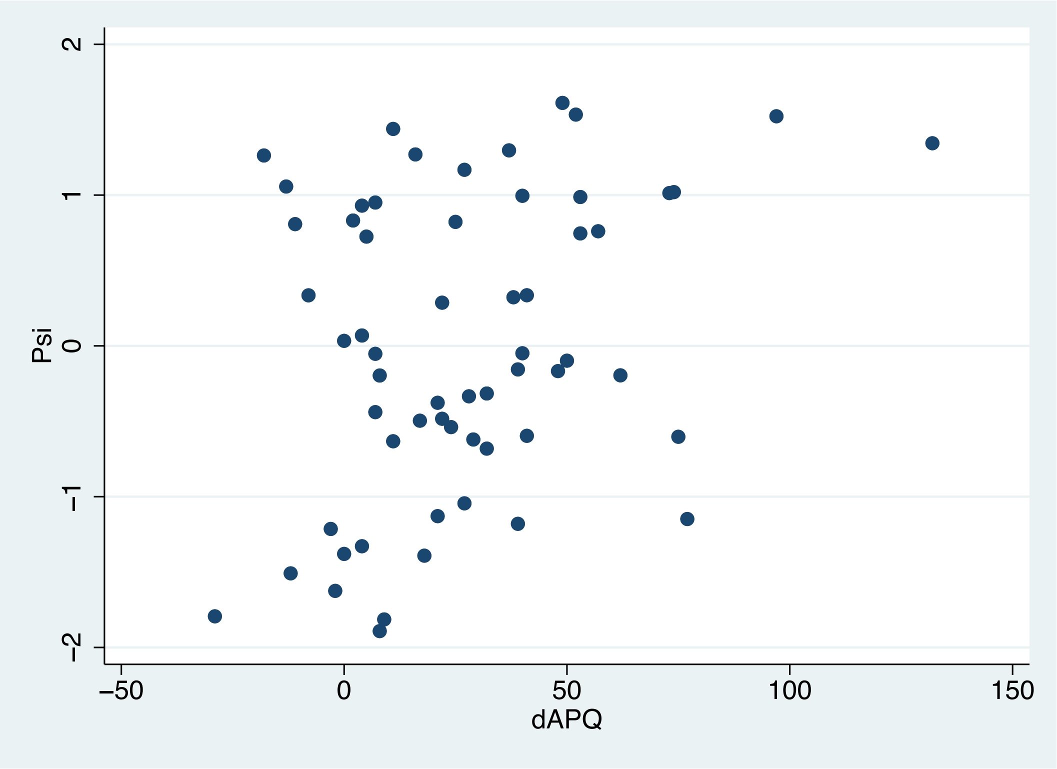

Figure E – Scatter diagram of change in APQ and Psi

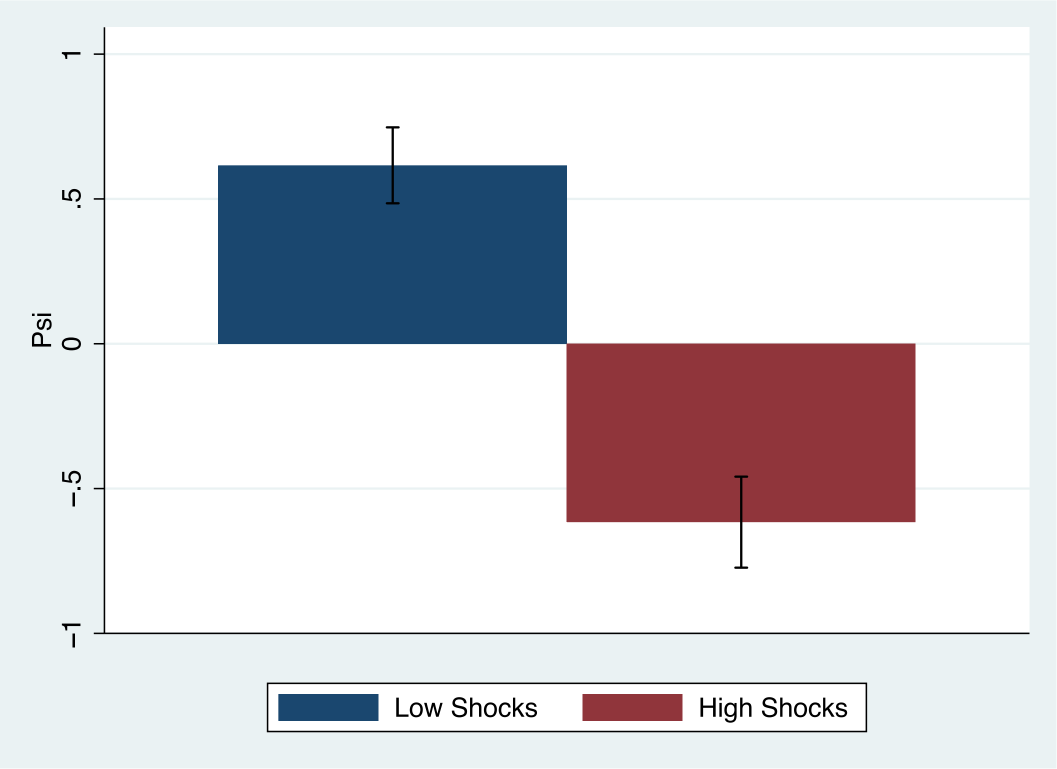

Figure F – Bar chart showing means and SEs of Psi by the Low and High Shock groups.

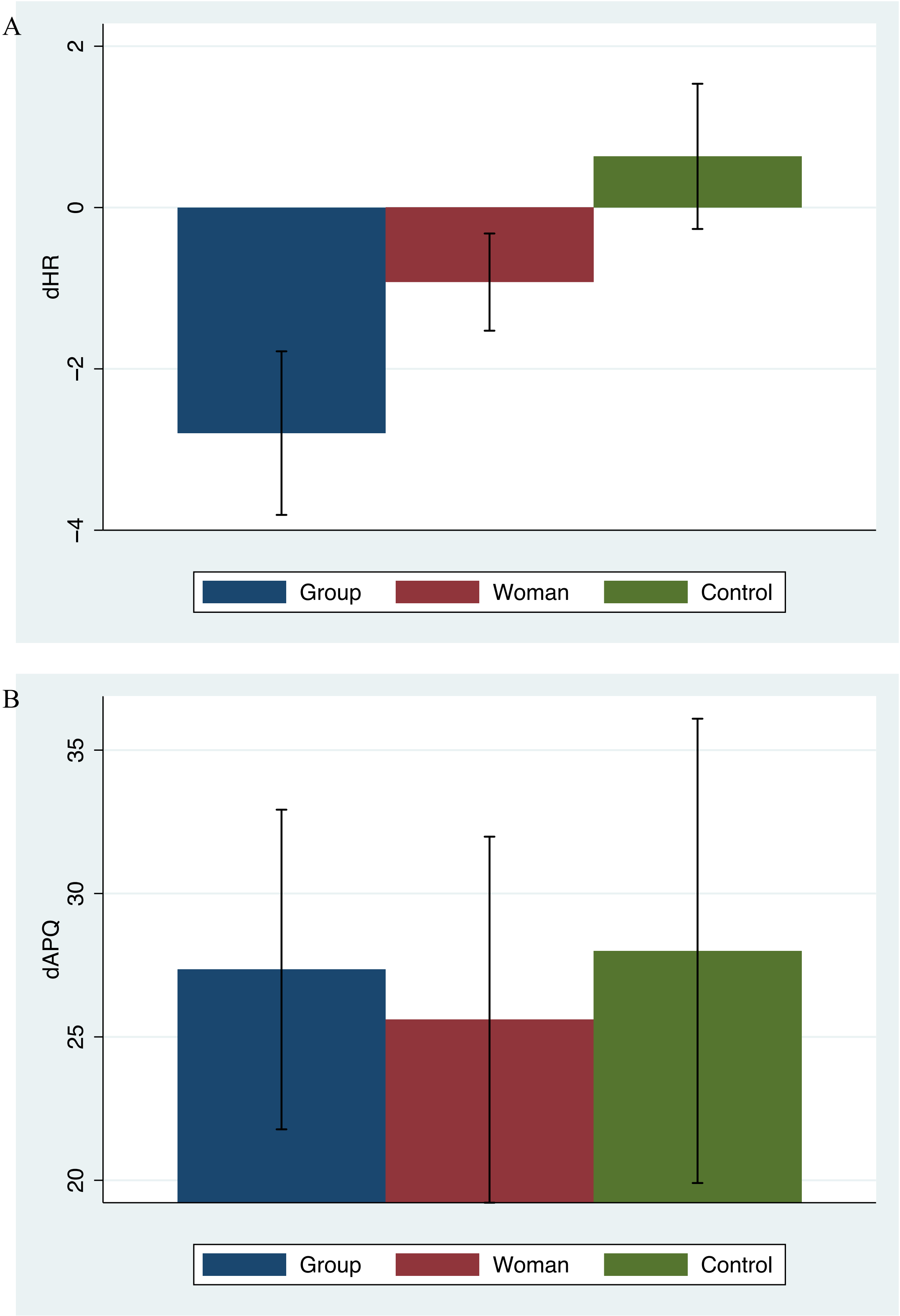

Figure G – Bar charts of means and standard errors of heart rate change (b.p.m.) and APQ change by Condition. (A) dHR (B) dAPQ.

Figure G shows the relationship between Condition and the change in heart rate (dHR) and the change in APQ (dAPQ). As shown in the main result the HR change is steeper for those in the Group condition than the Woman, and both are steeper than the Control condition. However, with respect to dAPQ there are no differences between the conditions.

We postulate a possible causal chain involving the change in heart rate (dHR), the change in APQ (dAPQ) reflecting interoception of participants' physiological state, plausibility and the and whether or not the number of shocks is high. This is shown in Figure H.

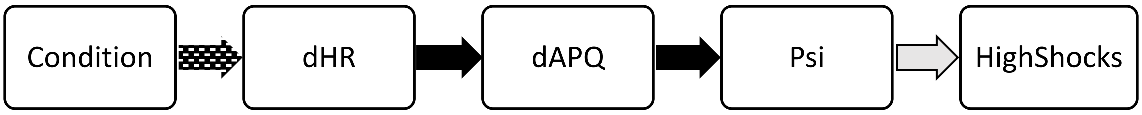

Figure H – A causal chain linking the condition (Woman, Group) to change in HR (dHR), the change in APQ (dAPQ), Plausibility (Psi) and High Shocks (nshocks > 15). The textured arrow indicates positive or negative relationship (Woman, Group can be different). The solid black arrows indicate positive correlation and the grey arrow a negative correlation.

This model states that an increase in HR is associated with an increase in interoception regarding subjective physiological state (dAPQ), which in turn is associated with higher plausibility, which is associated with a reduction in the number of shocks. Conversely, following the same chain, a decrease in HR compared with baseline results in an increase in the number of shocks. The Condition (Control, Woman, Group) are exogenous factors representing the experimental conditions.

This is formalised as the following model (with dHR and dAPQ standardized to mean 0 and SD 1).

$$\mu_{hr,i} = \beta_{hr,0} + \beta_{hr,1} W_i + \beta_{hr,2} G_i$$
$$dHR_i \sim normal(\mu_{hr,i}, \sigma_{hr})$$

$$\mu_{apq,i} = \beta_{apq,0} + \beta_{apq,1} dHR_i$$
$$dAPQ_i \sim normal(\mu_{apq,i}, \sigma_{apq})$$

$$\mu_{psi,i} = \beta_{psi,0} + \beta_{psi,1} dAPQ_i$$
$$Psi_i \sim normal(\mu_{psi,i}, \sigma_{psi})$$

$$\alpha_i = \beta_{hs,0} + \beta_{hs,1} Psi_i$$
$$HS_i \sim bernoulli\_logit(\alpha_i)$$

$$i = 1,2, \dots, n$$

......................(Eqn 1)

High shocks (HS_i) is 0 if the ith individual gave less than 15 shocks and 1 otherwise. $W_i = 1$ if the ith individual is in the Woman condition and 0 otherwise. $G_i = 1$ if the ith individual is in the Group condition and 0 otherwise. Control corresponds to $W_i = G_i = 0$.

Here *bernoulli_logit* (Stan library) is a standard binary logistic model suitable for modelling binary outcomes.

The prior distributions (weakly informative) are *normal*(0,10) for all the β parameters, and *Cauchy*(0,5) on the positive half-line for the standard deviation σ parameters.

The model fit was accomplished with 4000 iterations with 4 chains, there were no divergences, and all Rhat values were equal to 1.

The results are summarised in Table A. It can be seen that there are strong linear dependencies following the model in Figure H.

Table A - Summary of Posterior Distributions of the Model Parameters – Mean and SD are the mean and standard deviations of the posterior distributions, the 2.5 and 97.5 percentiles are shown. Prob>0 is the posterior probability that the parameter is > 0. No entry means that the parameter > 0 by definition. The data for dHR and dAPQ are standardised to mean 0 and SD 1.

				95% credible interval		Prob > 0
**parameter**	**Coeff. of:**	**mean**	**SD**	**2.5%**	**97.5%**	
**dHR**						
$\beta_{hr,0}$		0.41	0.22	-0.02	0.84	0.969
$\beta_{hr,1}$	*W*	-0.40	0.31	-1.01	0.20	0.096
$\beta_{hr,2}$	*G*	-0.88	0.32	-1.50	-0.24	0.003
σ_{hr}		0.97	0.10	0.80	1.18	
**dAPQ**						
$\beta_{apq,0}$		0.00	0.13	-0.26	0.25	0.496
$\beta_{apq,1}$	*dHR*	0.28	0.13	0.03	0.55	0.984
σ_{apq}		0.99	0.10	0.82	1.20	
**Psi**						
$\beta_{psi,0}$		0.01	0.13	-0.24	0.27	0.539
$\beta_{psi,1}$	*dAPQ*	0.32	0.13	0.07	0.58	0.992
σ_{psi}		0.99	0.10	0.82	1.21	
**HighShocks**						
$\beta_{hs,0}$		0.07	0.35	-0.61	0.77	0.570
$\beta_{hs,1}$	*Psi*	-1.86	0.46	-2.84	-1.03	0

Predicted posterior distributions on each of the three response variables were computed, and Table B gives the correlations between the means of these distributions and the observed values.

Following the same strategy as Supplementary Text S2 we use the 'leave-one-out' method for assessing the model. The results are shown in Table C. What is important is that the standard

errors are small compared with the estimates, and the effective number of parameters are in line with the actual number of parameters of the model.

Table B – Pearson correlations between means of the posterior predicted distributions and the observed values from the experiment (n = 58)

Variable	Pearson correlation	Conventional 95% confidence interval
dHR	0.36	0.109 to 0.564
dAPQ	0.28	0.027 to 0.504
Psi	0.31	0.061 to 0.530
HighShocks	0.63	0.450 to 0.767

Table C – Leave-one-out ('loo') results for the model. elpd_loo is a log-likelihood estimate of the goodness the predictive fit of the model, and p_loo is an estimate of the effective number of parameters (see also Text S2).

Model for	elpd_loo	SE	p_loo	SE
dHR	-81.9	4.8	3.7	0.6
dAPQ	-82.9	6.6	3.6	1.5
Psi	-82.3	3.5	2.3	0.3
HighShocks	-29.3	4.9	2.3	0.7

With the two end-points of the chain (Condition, HighShocks) fixed there are 6 possible model permutations. We analysed each of the 6 possible chains following the same model structure as above, and computed the leave-one-out statistic (elpd) and the correlations between the observed and means of the predicted posterior distributions for HighShocks.

Table D – Estimates of elpd differences and their standard errors for 6 models predicting HighShocks. The first row represents the model with the highest elpd, so that all entries in the elpd_diff column are differences from that model. The last two columns show the correlations between HighShocks and the means of the posterior predicted distributions for HighShocks (n = 58). Each model in column 1 is prefixed by $Condition \rightarrow$ and ends with $\rightarrow HighShocks$. The arrows → represent the proposed direction of causality.

Model	elpd_diff	SE	Pearson Correlation (r)	Conventional 95% confidence interval for r
$dHR \rightarrow dAPQ \rightarrow Psi$	0.000	0.000	0.63	0.449 to 0.767
$dAPQ \rightarrow dHR \rightarrow Psi$	-0.104	0.031	0.64	0.453 to 0.769
$Psi \rightarrow dAPQ \rightarrow dHR$	-12.651	4.973	0.11	-0.157 to 0.354
$dAPQ \rightarrow Psi \rightarrow dHR$	-12.748	4.979	0.11	-0.156 to 0.356
$dHR \rightarrow Psi \rightarrow dAPQ$	-13.083	4.987	0.01	-0.272 to 0.245
$Psi \rightarrow dHR \rightarrow dAPQ$	-13.151	4.993	-0.09	-0.343 to 0.169

The results are shown in Table D. The model with the highest elpd is that shown in Figure H. There is a small reduction in elpd for the model where the order of *dAPQ* and *dHR* are swapped, but the SE of the reduction is small compared to the size. However, the correlations between the observed and predicted values are the same. All other models have a much lower elpd and small correlations.

Figure G shows that there is a clear relationship between condition and dHR but no such relationship between condition and dAPQ. Therefore it seems reasonable to conclude that the preferred model is that shown in Figure H.

Regarding dHR, the Control condition corresponds to the parameter $\beta_{hr,0}$, the Woman condition to $\beta_{hr,0} + \beta_{hr,1}$ and the Group condition to $\beta_{hr,2}$. The posterior distributions are shown in Figure I. It can be seen that Group is associated with a decrease in dHR, Control with an increase, and no change in the case of the Woman condition.

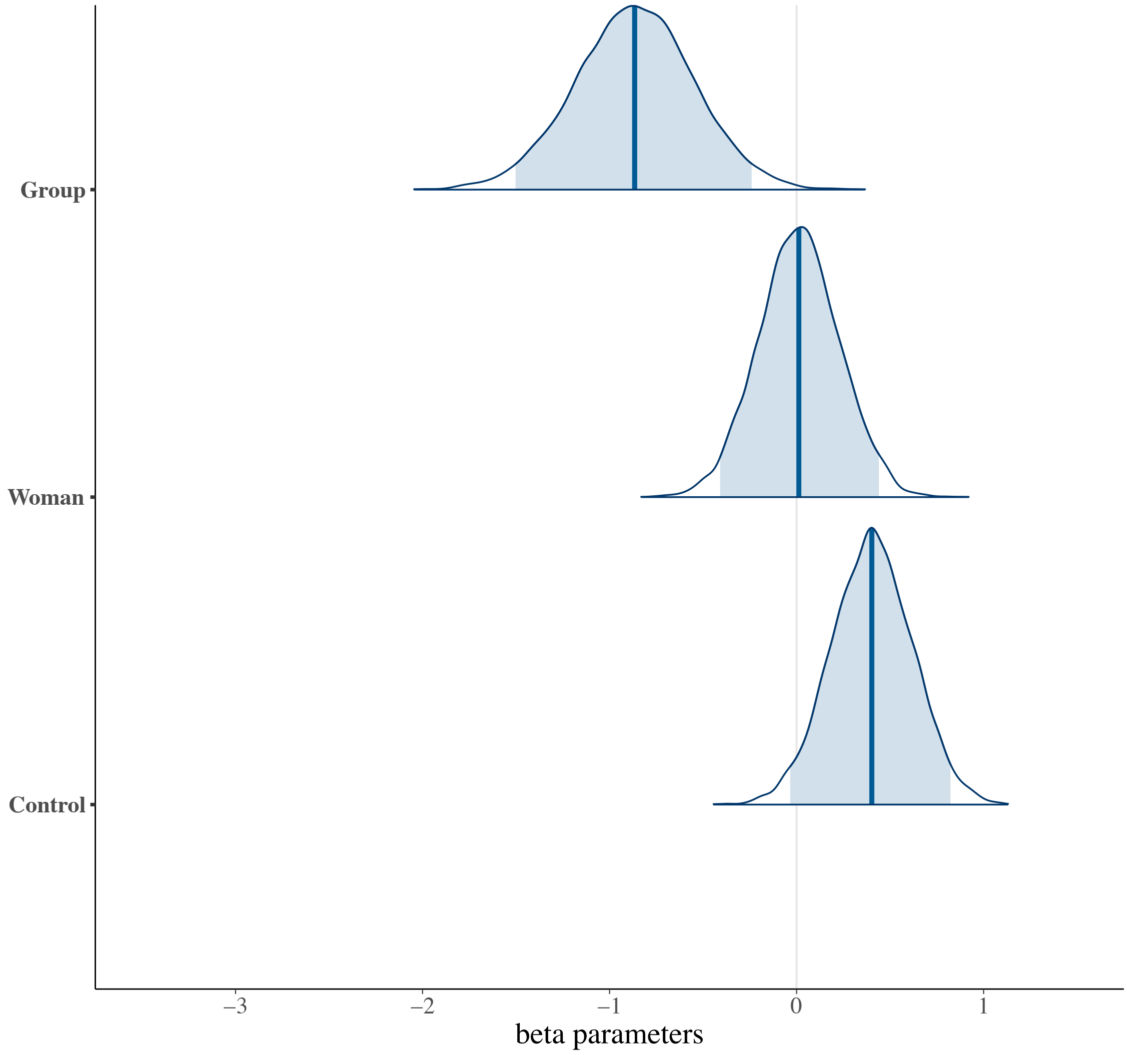

Figure I – posterior distributions of the parameters for Control ($\beta_{hr,0}$), Woman ($\beta_{hr,0} + \beta_{hr,1}$) and Group ($\beta_{hr,2}$) from Eqn 1. The thick vertical lines are the means of the distributions and the shaded areas are 95% credible intervals.

An Embodied Perspective as a Victim of Sexual Harassment in Virtual Reality Reduces Action Conformity in a Later Milgram Obedience Scenario

Solène Neyret, Xavi Navarro, Alejandro Beacco, Ramon Oliva, Pierre Bourdin, Jose Valenzuela, Itxaso Barberia, Mel Slater

Supplementary Text S4
Qualitative results

We present here two word-clouds showing the frequency of words spontaneously used by the participants to describe their experience during the second exposure to the verbal harassment scenario (answering to the question: *How did you feel during the second part of the experience?*). The word clouds were generated using the following method: we ran a frequency test using the software for qualitative analysis Nvivo 12[1]. Each word that was repeated at least twice (within one condition or between conditions) was selected for the word cloud. We then ran a frequency analysis for each word in each condition (Table A). We then checked that each word counted was used only once by each participant to avoid artificial effect of frequency when one participant was using the same word several times. Nvivo allows the grouping of close synonyms together, therefore in some cases some words with similar meaning are counted as a repetition of the same word, for instance the word "fear" was counted as a repetition of the word "afraid" (the detail of each of those cases is presented in Table A).

Table A - Words selected for the creation of both word-clouds, the frequency is presented for each word in each condition. Words are classified here from the most frequently to the least frequently used in the woman condition. A translation of each word to English is presented in the second column.

Words (Spanish)	Translation	Words frequency Group condition	Words frequency Woman condition
Acosado	Harassed	0	5
Incómodo	Uncomfortable	2	5
Espectador	Spectator	1	3
Imbécil(es), capullos	Idiot(s)	0	3
Molestando	Bothering	0	3
Nervioso, intranquilo	Nervous, worried	0	3
Aburrido	Annoyed	0	2
Asustado, miedo	Afraid, fear	0	2

[1] https://www.qsrinternational.com/nvivo/nvivo-products/nvivo-12-plus

Impotente	Powerless	1	2
Levantarme	Stand up	0	2
Reflexionar	Reflect on	0	2
Violento	Violent	0	2
Observando	Observing	2	1
Película	Movie	1	1
Calmado, cómodo	Calm, Comfortable	2	0
Extraño	Strange	3	0
Pena	Pity	2	0

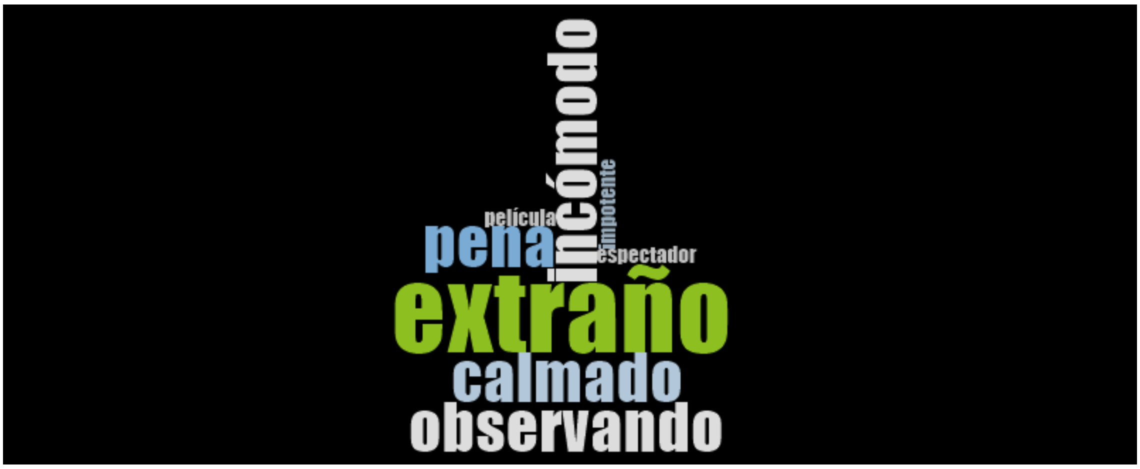

Figure A - word cloud representing the frequency of words used by the participants in the Group condition after the second exposure to the verbal harassment scene

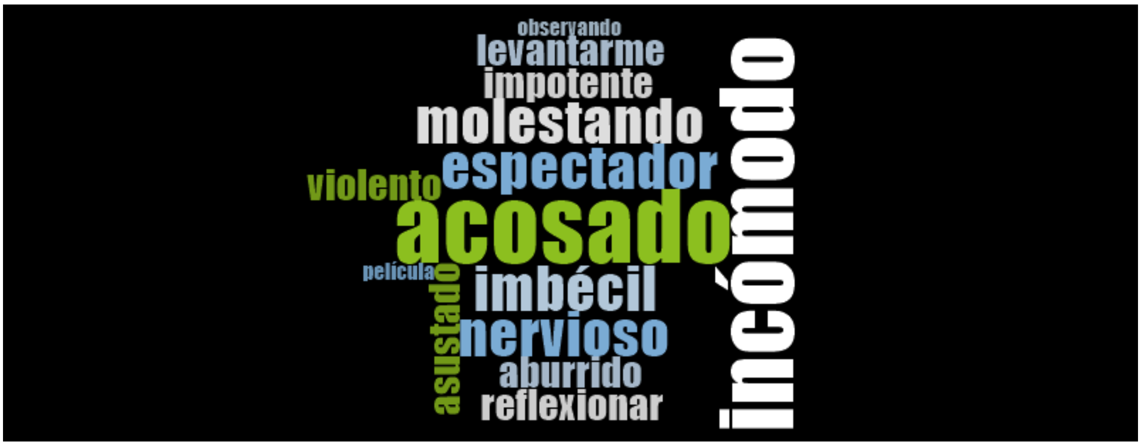

Figure B - word cloud representing the frequency of words used by the participants in the Woman condition after the second exposure to the verbal harassment scene

The word "acosado" (*harassed*) was used only in the woman condition, showing that the effect we wanted to create was successful. 5 participants out of 20 (in the Woman condition) used this word to spontaneously describe their experience inside the virtual scene (25%). The word "incómodo" (*uncomfortable*) was used in both conditions but with more frequency in the woman condition (10% in the Group condition and 25% in the woman condition). The

qualification "Imbécil(es)" (*idiot(s)*) describing the avatar(s) harassing the woman, was used only in the woman condition showing that participants in the Group condition did not get this negative perception of the male avatars. Participants in the Group condition reported more diverse sensations, it was difficult to find words repeated more than 2 or 3 times between participants. The word repeated with greater frequency was the word "extraño" (*strange)*. For the participants in the Group condition there was a small effect of feeling "outside" the scene (*observing, spectator, movie*), only 2 participants reported that they were feeling "*pity*" for the victim. More surprisingly, 2 participants reported feelings of "*calmness*" during the harassment scene.

An Embodied Perspective as a Victim of Sexual Harassment in Virtual Reality Reduces Action Conformity in a Later Milgram Obedience Scenario

Solène Neyret, Xavi Navarro, Alejandro Beacco, Ramon Oliva, Pierre Bourdin, Jose Valenzuela, Itxaso Barberia, Mel Slater

Supplementary Text S5

Presence and Body Ownership

Presence

Presence is the illusion of being in the virtual place (Place Illusion, PI), and also the extent to which the situation and events seemed to be really happening (Plausibility Illusion, Psi). This is covered in the questionnaire by two variables:

PI: I had the sensation to be on the terrace.
Psi: I had the sensation that the conversation was really happening.

The responses were on the scale -3 (complete disagreement) to +3 (complete agreement) and the questions were administered after each phase. Figure A shows the results for PI. It is clear that the place illusion was high under all conditions and phases, and not different between the phases. Figure B shows the similar results for Psi.

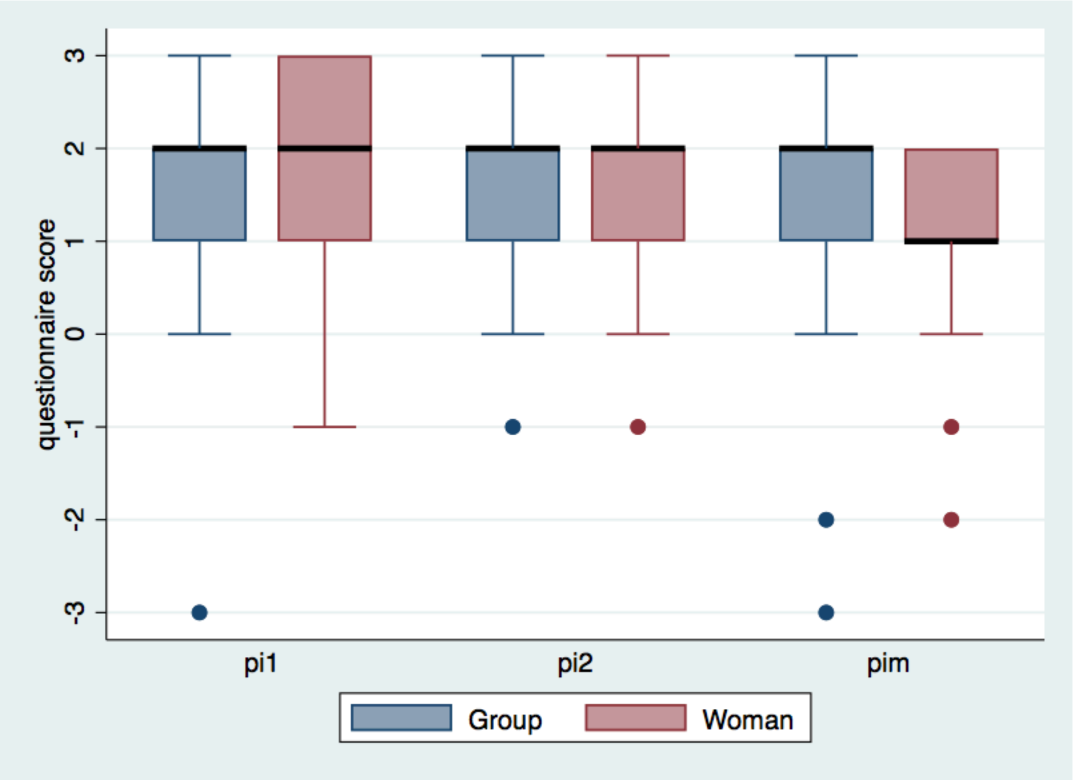

Figure A - Box plots of PI by phase 1 and 2 and condition. PIm refers to PI during the Obedience (Milgram) experiment. The horizontal thick lines are the medians, the boxes are the interquartile ranges (IQR), the whiskers extend from max(median - 1.5*IQR, smallest value) to min(median + 1.5*IQR, largest value). Scores outside this range are shown individually.

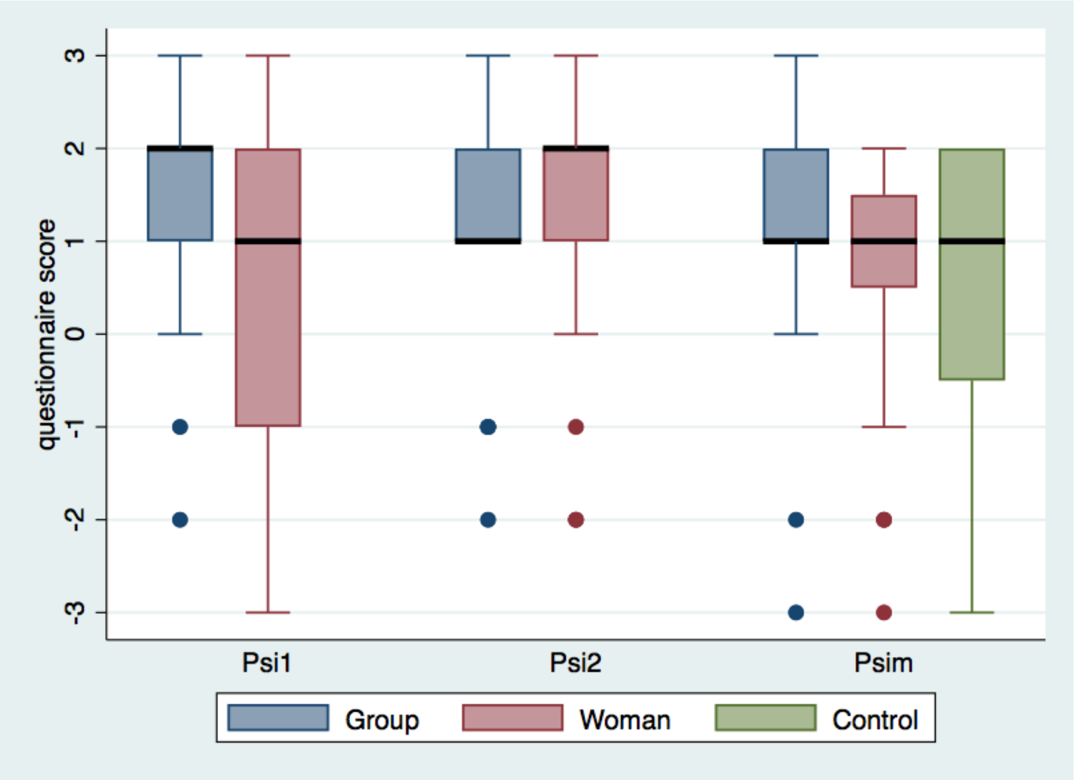

Figure B - Box plots of Psi by phase 1 and 2 and condition. Psim refers to Psi during the Obedience (Milgram) experiment.

Body Ownership

An important response variable in the Bar scenario was the extent to which participants had the perceptual illusion that the virtual body that they embodied was their body. For this purpose we had administered the following questions immediately after each virtual exposure:

mirror: I had the feeling that the virtual body I saw when I looked towards the mirror was my body.

down: I had the feeling that the virtual body I saw when I looked down was my body.

Each of these were scored on a -3 to +3 scale, where -3 signifies complete disagreement and 3 complete agreement. These questions were given after Phase 1 and Phase 2 where mirror1 is the score after phase 1, mirror2 after phase 2 and similarly for down.

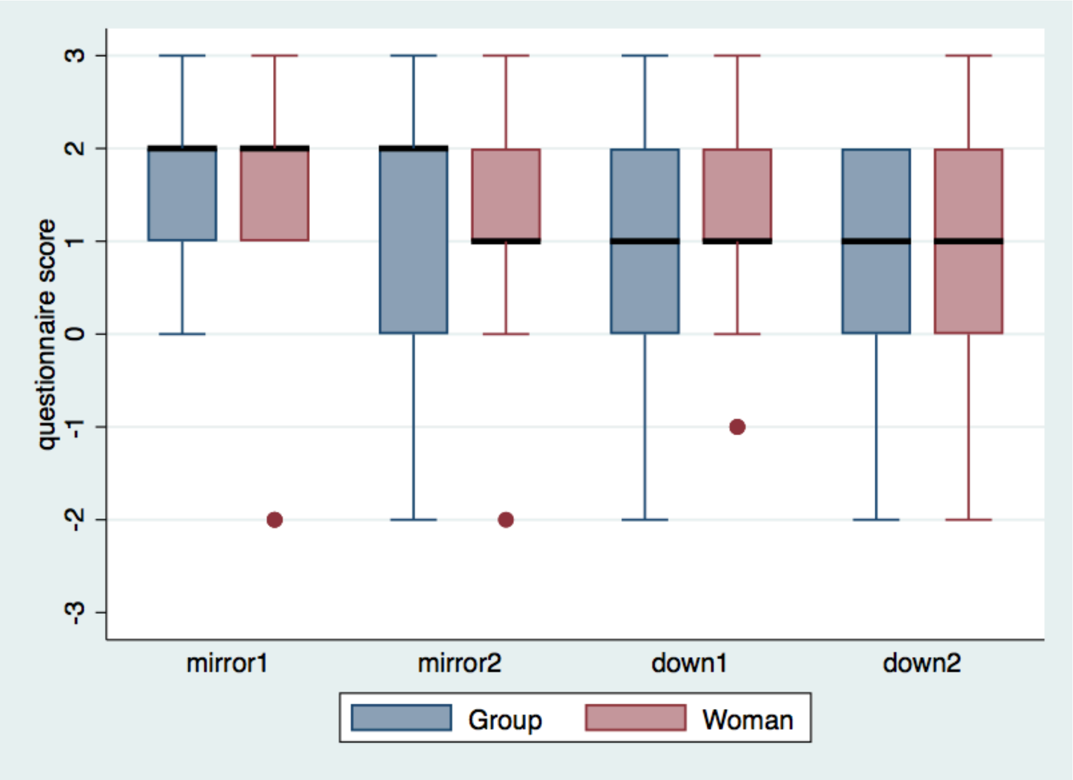

Figure C - Box plots of body ownership by phase 1 and 2 and condition.

Figure C shows the box plots of the two questionnaire scores by phase and condition. It is clear that overall the body ownership scores were high (for example, all the interquartile ranges are above the 0 score, and all the medians are 1 or 2 out of the maximum score of 3). Most important, and in line with other findings, the level of subjective body ownership does not differ whether the participants are embodied in the male or female body, and also do not vary between the two phases.

An Embodied Perspective as a Victim of Sexual Harassment in Virtual Reality Reduces Action Conformity in a Later Milgram Obedience Scenario

Solène Neyret, Xavi Navarro, Alejandro Beacco, Ramon Oliva, Pierre Bourdin, Jose Valenzuela, Itxaso Barberia, Mel Slater

Supplementary Text S6

Differences Between Low and High Shock Groups

Here we consider the Low shock group to be those who gave 14 or less shocks, the remainder being the High shock group. (It makes no perceptible difference if 12 is used as the cut-off rather than 15, but we follow the findings in Figure 8A, main text).

1. The NEO personality scores

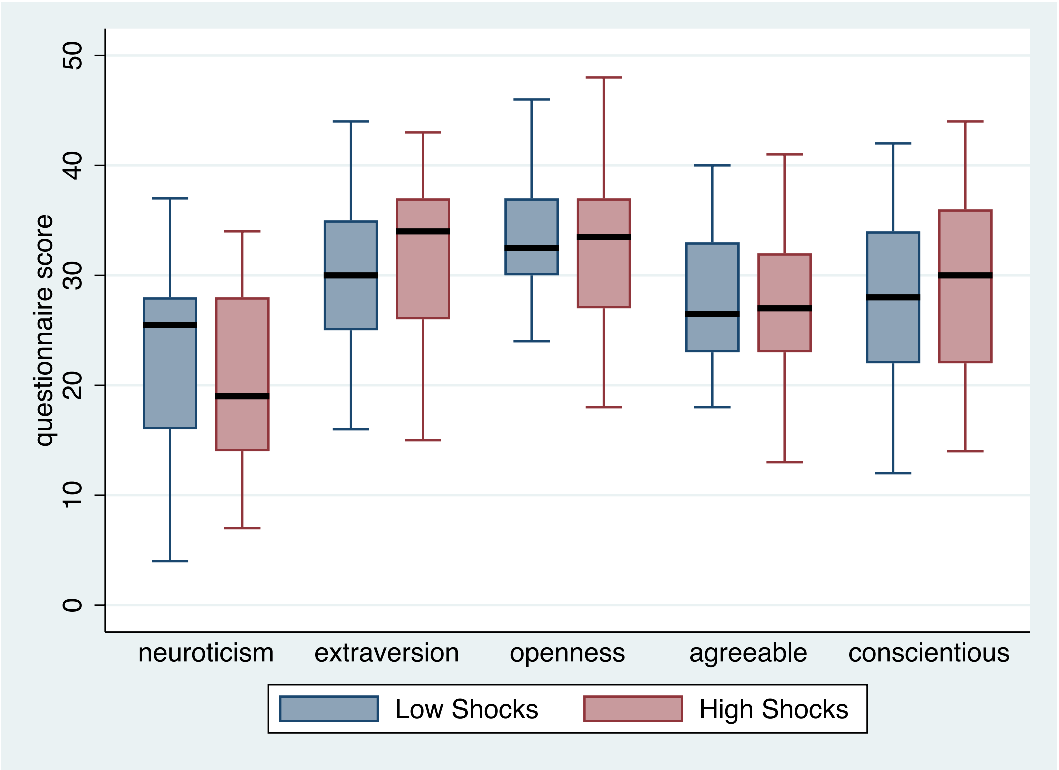

Figure A – Box plots for the NEO personality scores.

Figure A shows the box plots for the NEO personality scores, and it is clear that there are no differences between those who gave low or high shocks.

2. Sexual harassment

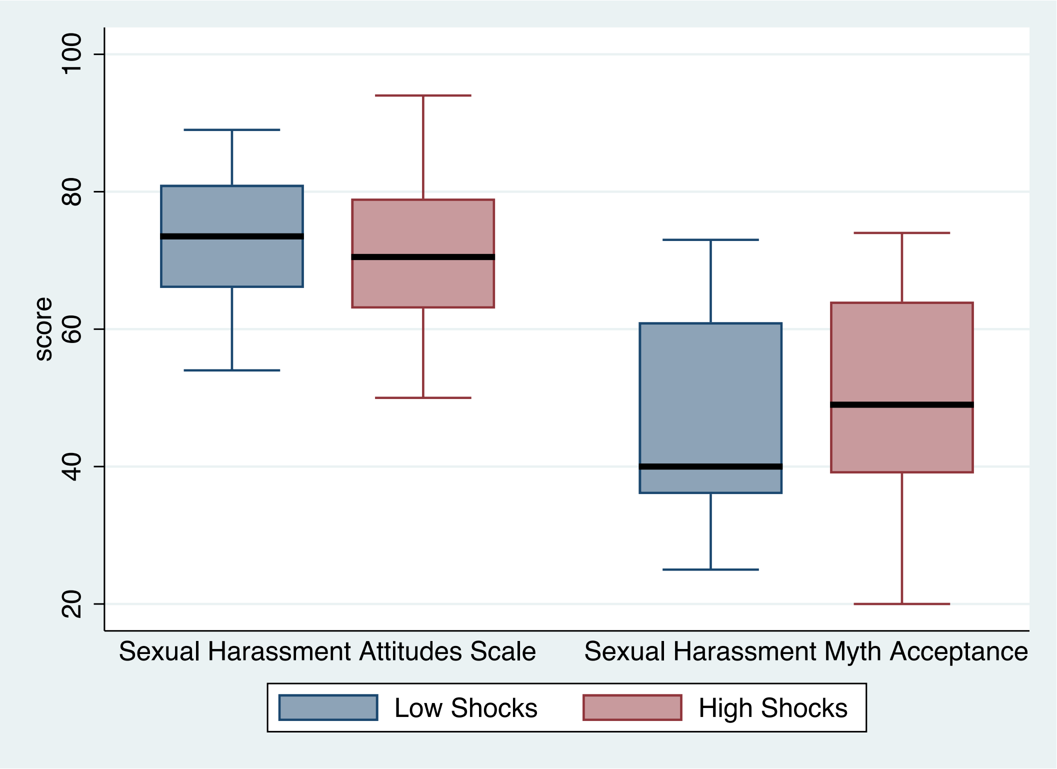

Figure B – Box plots for the harassment scores.

Figure B shows the box plots for the sexual harassment questionnaires, and it is clear that there are no differences between the conditions.

3. Place Illusion

Place Illusion was measured by the following questions. The questions are based on those in Steed, et al. [1] and many previous papers.

Table A – Questions for Place Illusion.

**Variable**	**Question**
there	Please rate your feeling of being in the training room situation with the following scale from -3 to +3 (in which +3 represents the feeling you usually have when you're in a place).
real	To what extent did you feel at certain times during the experience that the training room was the reality for you?<br>(-3 not at all, 3 all the time)
beenthere	When you think about your experience, do you remember the situation in the training room as if it were some images that you have seen or as if it were a place where you have been?<br>(-3 images, 3 place been)
intrainingroom	During the experience, what has been stronger, the feeling of being in the training room or the feeling of being in the real world of the virtual reality laboratory?<br>(-3 laboratory, 3 virtual training room)

Figure C shows the box plot of the results. Again it is clear that there were no salient differences between the low and high shock groups.

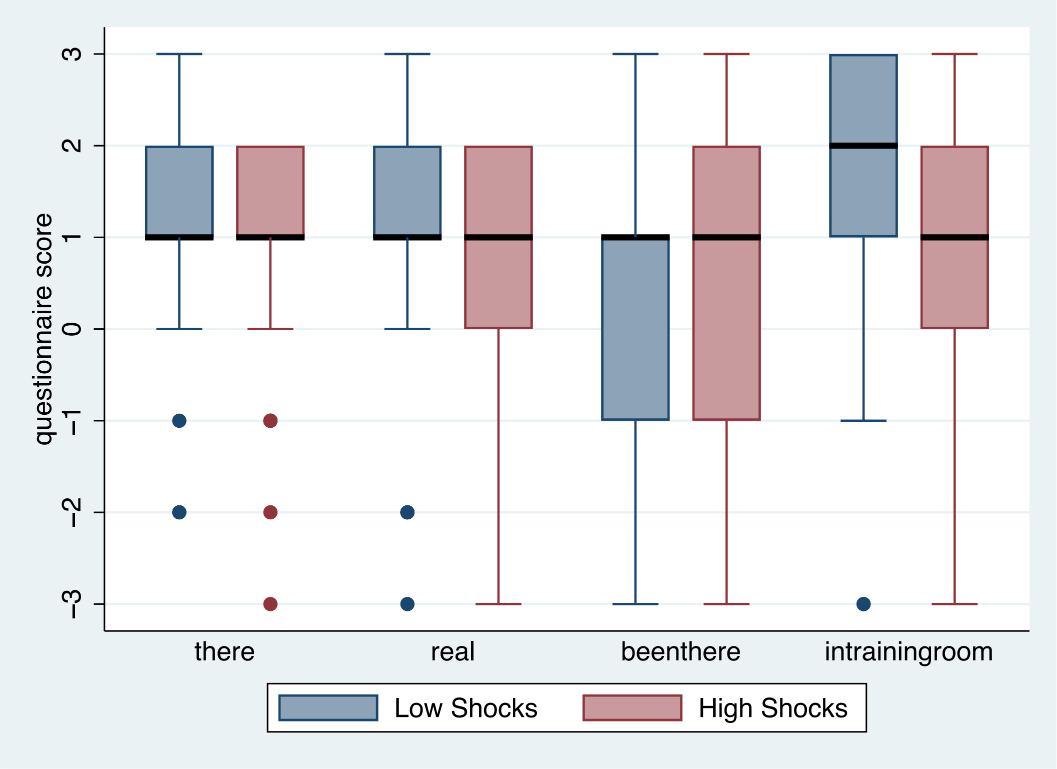

Figure C – Box plots for the Place Illusion scores.

References

1 Steed, A., Pan, Y., Watson, Z. & Slater, M. 'We Wait'-The Impact of Character Responsiveness and Self Embodiment on Presence and Interest in an Immersive News Experience. *Frontiers in Robotics and AI* **5**, 112 (2018).

An Embodied Perspective as a Victim of Sexual Harassment in Virtual Reality Reduces Action Conformity in a Later Milgram Obedience Scenario

Solène Neyret, Xavi Navarro, Alejandro Beacco, Ramon Oliva, Pierre Bourdin, Jose Valenzuela, Itxaso Barberia, Mel Slater

Supplementary Text S7

Qualitative Results Related to Ethics

A. Why did participants continue giving shocks?

At the end of the Shocks scenario, we collected the subjective reports of all our participants in the form of a qualitative questionnaire asking "*If you wanted to stop, why did you continue*". The results of the participants in the Group condition shows two main tendencies for finishing the procedure:

- Thinking that they were not really hurting anyone (50%)
- Duty of finishing the procedure and curiosity about the end of the experiment (50%)

Examples of answers from participants in the Group condition to the question: "*If you wanted to stop, why did you continue*":

"Because I thought it was a virtual reality scenario, and that in reality I was not hurting anyone in real life. Likewise, even though the woman was complaining she did not show any sign of being burnt, her aspect seemed quite 'normal'".

"In that moment I thought it was not real".

"Out of curiosity about the experiment".

"Because deep down, I knew that it was not real and I wanted to complete the experience, and since it was a virtual experience, the only way to stop was interrupting the experiment (the real one)."

" Because it was not real and I had the sensation I could not really interact with the (virtual) experimenters:"

"Because I was conscious that it was not real, and to reach the end of the experiment."

" To finish the experiment"

"Because it was not real".

“Because if not, the experiment was not making any progress.”

“Because my fellows were telling me to continue and that they had already made an agreement with her.”

B. Follow up session for participants

Follow-up sessions happened one month after the end of the completion of the study, participants were called back to the laboratory via personal email, they were immersed in the virtual environment of the Bar scenario and embodied as the female in order to perceive the harassment scene from the “victim perspective”, the total length of the session was 20 minutes and they were paid 5 euros for their time.

Some examples of the feedback we received to that long-term email from participants in the Group condition who could not come to the follow up session and did not get embodied in the woman are reported here:

“I searched on the internet about the original study on which your experiment was based, since it was very interesting. And it made me reflect a bit upon how we function in an organized society”

“I though in possible futures, when this technology will reach massive consumption”

Another participant who was in the Woman condition answered to the follow-up email:

Maybe if I find myself in such a situation in reality, I should do something.
